# Dynamic modeling and analysis of the rotor–stator coupling system of a coaxial contra-rotating gearbox

**DOI:** 10.1038/s41598-023-49387-5

**Published:** 2023-12-13

**Authors:** Donglin Zhang, Ji Cui, Rupeng Zhu, Miaomiao Li

**Affiliations:** 1 School of Intelligent Manufacturing, Nanjing Vocational College of Information Technology, Nanjing, 210023 China; 2 National Key Laboratory of Science and Technology on Helicopter Transmission, Nanjing University of Aeronautics and Astronautics, Nanjing, 210016 China

**Keywords:** Aerospace engineering, Mechanical engineering

## Abstract

In this paper, a main gearbox using an encased differential gear train to achieve coaxial contra-rotating is considered, and a dynamic modeling method of the rotor–stator coupling system of the gearbox based on box model updating is introduced. The transverse torsional dynamic model of the gear transmission subsystem is established based on the lumped parameter method. The finite element model of the box is updated according to the modal test data, and the reduced dynamic parameters of the box are obtained. According to the displacement coordination condition, the dynamic model of the rotor–stator system of the gearbox is established. The vibration response of the transmission components with or without the coupling box is calculated by numerical integration, and the response of the box caused by the dynamic support reaction force is analyzed by the finite element method. The results show that the vibration peak and fluctuation range of the transmission parts with coupling box are smaller than those without coupling box. The box response at the support of the input bevel gear pair is large, while that at the support of the output shaft is small.

## Introduction

The coaxial twin-rotor helicopter has a pair of coaxial counter rotating rotors, which can provide more and symmetrical lift. When the tail propeller is used to provide thrust, it can realize high-speed flight, which has incomparable advantages over single-rotor helicopters^1,2^. However, to realize the counter rotation of the dual rotors, there are two reverse branches in the main transmission system, which makes the power flow more complex. The inner and outer output shafts support each other through intermediate bearings. These factors make the vibration of the gearbox caused by the dynamic meshing force and supporting force very prominent, so it is necessary to study its dynamic characteristics^3^.

Many studies have focused on the vibration and noise of the gearbox. The dynamic modeling methods of gear transmission systems mainly include the lumped parameter method, finite element method, and hybrid modeling method. Most of the early research focused on lumped-parameter models^4–6^. Lin and Parker^4^ established a lumped-parameter rotational translational model of planetary gears and studied the natural frequencies and vibration modes of the system based on the model. They also analyzed the sensitivity of natural frequency and vibration modes to system parameters^5^ and the rules of eigenvalue veering in planetary gears^6^. Stringer et al.^7^ presented a finite element model that includes the shaft, gear couplings and gear mesh of a helicopter transmission and discussed the natural frequencies and mode shapes of the system.

Considering the geometric eccentricity of the gear meshing and the flexibility of the bearing, Zhang^8^ modeled the rotating shaft of the system as a Timoshenko beam element and established the three-dimensional dynamic model of a multishaft helical gear rotor system. The forced response of the system to geometric eccentricity and rotor mass imbalance is analyzed. Zhang et al.^9^ established a coupled lateral-torsional-axial vibration model of a multistage planetary gear system using the shafting element method. The natural frequency and vibration mode of the system were obtained by using the model, and the influence of stiffness parameters on the natural characteristics was studied. Abousleiman and Velex^10,11^ established 3D finite element models of the ring gear and carrier that can consider the deformation, connected the lumped parameter model of gear elements and shaft elements based on the modal condensation technique, and established a model that can simulate the three-dimensional dynamic characteristics of the planetary spur gear and helical gear with deformable parts.

Concli^12^ calculated the stiffness of the bearing and the gear mesh by finite element simulation, established a lumped parameter model with 18 degrees of freedom of the planetary gearbox, and verified the correctness of the predicted frequency by the model through experiments. Ericson and Parker^13^ obtained the modal characteristics and vibration response of two spur planetary gears by using experimental modal analysis techniques and compared them with the results obtained by the lumped parameter model and finite element model. They also further analyzed the variation law of natural frequencies^14^ with parameters by numerical methods.

To consider the influence of the box on the vibration characteristics of the gearbox system, it is necessary to establish the dynamic model of the coupling transmission subsystem and the box subsystem. Ambarisha^15^ established a helical gearbox model considering two different housing models, the full finite element model and the reduced model of condensed stiffness and mass matrices obtained by the component modal synthesis (CMS) method, and proved the rationality of using the reduced housing model by comparing the calculation results of the two models. Wang^16^ modeled the carrier, housing and bedplate as flexible bodies and other components as rigid bodies, established a rigid-flexible coupling dynamic model for a wind turbine gearbox, and studied the influence of gear tooth modification on dynamics. Lu^17^ used the substructure method to extract the mass and stiffness parameters of the box and established the coupling dynamic model considering the gear subsystem and the box subsystem. The dynamic characteristics of the continuous shearer’s gearbox are studied, and a load vibration test is carried out. The analysis results showed that the dynamic model coupled with the box is closer to the actual situation. Based on the theories of structural dynamics and system dynamics, Liu^18–20^ established an improved numerical model of a double-helical gearbox by a hybrid user-defined element method (HUELM) combined with TCA and LTCA methods. The model is composed of four special finite elements: gear pair element, bearing element, three-dimensional flexible shaft element, and shell element, which overcomes the shortcomings that the lumped parameter method cannot calculate the box response and the large amount of calculation in finite element method analysis. The dynamic response of the shell is obtained by the test, which verifies the correctness of the model.

However, little research has been done on the dynamic modeling and analysis of the rotor–stator coupling system of the coaxial contra-rotating dual-output gearbox combined with the box modal test and model updating. In this paper, the rotor–stator coupling dynamic modeling and dynamic response characteristics of a coaxial gearbox considering box updating are studied.

## Dynamic model of the transmission system

**Figure 1:**
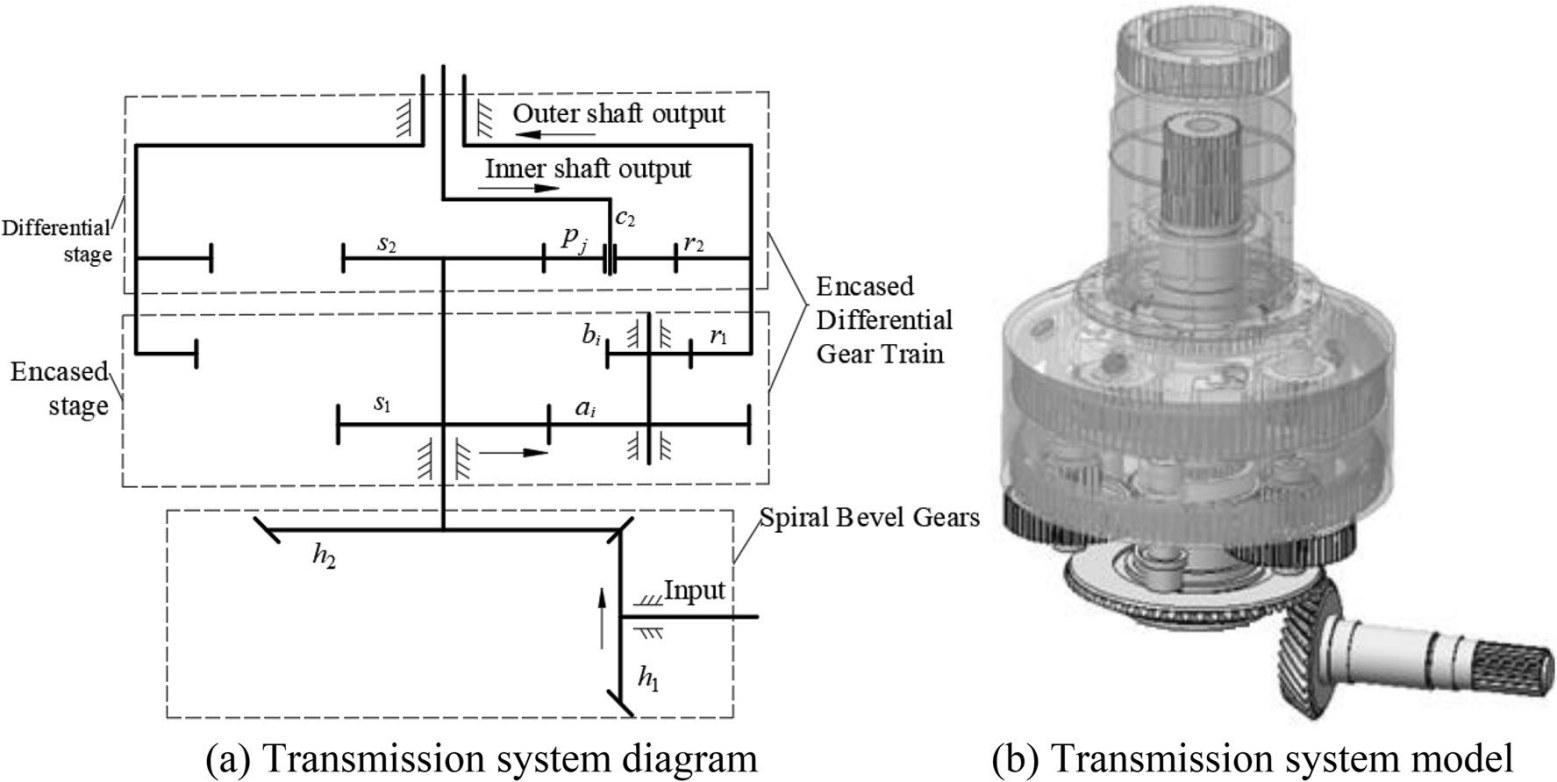
Transmission system of coaxial contra-rotating main gearbox.

The transmission system of the coaxial main gearbox consists of a spiral bevel gear pair and an encased differential gear train, as shown in Fig. 1. The encased differential gear train can be divided into two parts: the encased stage composed of sun gear *s*_1_, stepped planets *a*_*i*_, *b*_*i*_ and ring gear *r*_1_, and the differential stage composed of sun gear *s*_2_, planet *p*_*j*_, ring gear *r*_2_ and carrier *c*_2_.

When the input power is transmitted to the encased differential gear train through the spiral bevel gear pair, it is divided into two paths: one part is transmitted from sun gear *s*_1_ to ring gear *r*_1_, and the other part is transmitted from sun gear *s*_2_ to carrier *c*_2_ and ring gear *r*_2_ through the differential stage. The transmission power of the carrier *c*_2_ is output by the inner shaft *c*_*s*_, and the power of the ring gear *r*_1_ and *r*_2_ is output by the outer shaft *r*_*s*_ after confluence.

Through the proper parameter design, as shown in Table 1, the inner and outer output shafts can rotate in opposite directions at the same speed. In the system, the number of planets *p* is *N*, and the number of stepped planets *ab* is *M*.

**Table 1:** The parameters of the gearbox.

	Teeth number	Module (mm)	Mass (kg)	Inertia (kg m^2^)	Pressure (°)	Helix angle (°)
*h*1	29	5.5	12.7	0.0253	20	35
*h*2	56	5.5	13.0	0.2150	20	35
*s*1	57	2.75	7.76	0.0267	20	0
*a*	54	2.75	3.14	0.0106	20	0
*b*	18	3.5	1.286	6 × 10^–4^	20	0
*r*1	107	3.5	10.51	0.9280	20	0
*s*2	38	4	6.12	0.0205	20	0
*p*	25	4	2.0	0.0035	20	0
*r*2	88	4	1.72	1.7224	20	0

### Coordinates and nomenclature

**Figure 2:**
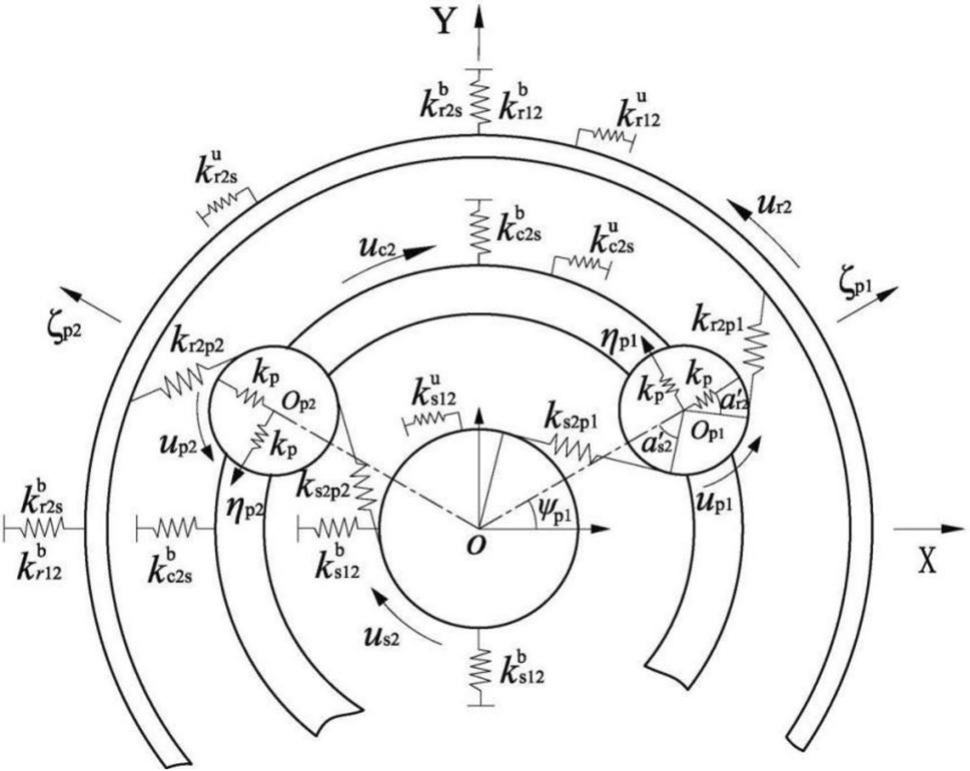
Dynamic model of the differential stage.

The lumped parameter dynamic model of the differential stage gear train is established based on the fixed coordinate system OXY, whose origin is the theoretical installation center of sun gear *s*_2_, as shown in Fig. 2. The local coordinate system $O_{{{\text{p}}j}} \zeta_{{{\text{p}}j}} \eta_{{{\text{p}}j}}$ is fixed on the carrier and rotates with it at a constant speed, and its origin is located at the theoretical installation center $O_{{{\text{p}}j}}$ of the *j*-th planet.

When the position coordinate of the centroid of planet *p*_*j*_ in the local coordinate system is ($\zeta_{{{\text{p}}j}} ,\eta_{{{\text{p}}j}}$), the distance $OO_{{{\text{p}}j}}^{\prime }$ in the coordinate system OXY can be expressed as:

$$ \begin{aligned} OO_{{{\text{p}}j}}^{\prime } & = OO_{{{\text{p}}j}} + O_{{{\text{p}}j}} O_{{{\text{p}}j}}^{\prime } = (r_{o} \cos \psi_{{{\text{p}}j}} + \zeta_{{{\text{p}}j}} \cos \psi_{{{\text{p}}j}} - \eta_{{{\text{p}}j}} \sin \psi_{{{\text{p}}j}} )i \\ & \;\;\; + (r_{o} \sin \psi_{{{\text{p}}j}} + \zeta_{{{\text{p}}j}} \sin \psi_{{{\text{p}}j}} + \eta_{{{\text{p}}j}} \cos \psi_{{{\text{p}}j}} )j = xi + yj \\ \end{aligned} $$

According to the coordinate transformation rules, the absolute acceleration components of the planet *p*_*j*_ in the $O_{{{\text{p}}j}} \zeta_{{{\text{p}}j}}$ and $O_{{{\text{p}}j}} \eta_{{{\text{p}}j}}$ directions are shown as follows^21^:

1 $$ a_{{{\text{p}}j}} = \left[ {\begin{array}{*{20}c} {a_{\zeta j} } \\ {a_{\eta j} } \\ \end{array} } \right] = \left[ {\begin{array}{*{20}c} {\cos \psi_{{{\text{p}}j}} } & {\sin \psi_{{{\text{p}}j}} } \\ { - \sin \psi_{{{\text{p}}j}} } & {\cos \psi_{{{\text{p}}j}} } \\ \end{array} } \right]\left[ {\begin{array}{*{20}c} {\ddot{x}} \\ {\ddot{y}} \\ \end{array} } \right] $$

There are

2 $$ a_{\zeta j} = \ddot{\zeta }_{{{\text{p}}j}} + 2\omega_{{{\text{c2}}}} \dot{\eta }_{{{\text{p}}j}} - \omega_{{{\text{c2}}}}^{{2}} \zeta_{{{\text{p}}j}} - \omega_{{{\text{c2}}}}^{{2}} r_{{{\text{c2}}}} $$

3 $$ a_{\eta j} \; = \;\ddot{\eta }_{{{\text{p}}j}} - 2\omega_{{{\text{c2}}}} \dot{\zeta }_{{{\text{p}}j}} - \omega_{{{\text{c2}}}}^{{2}} \eta_{{{\text{p}}j}} $$

**Figure 3:**
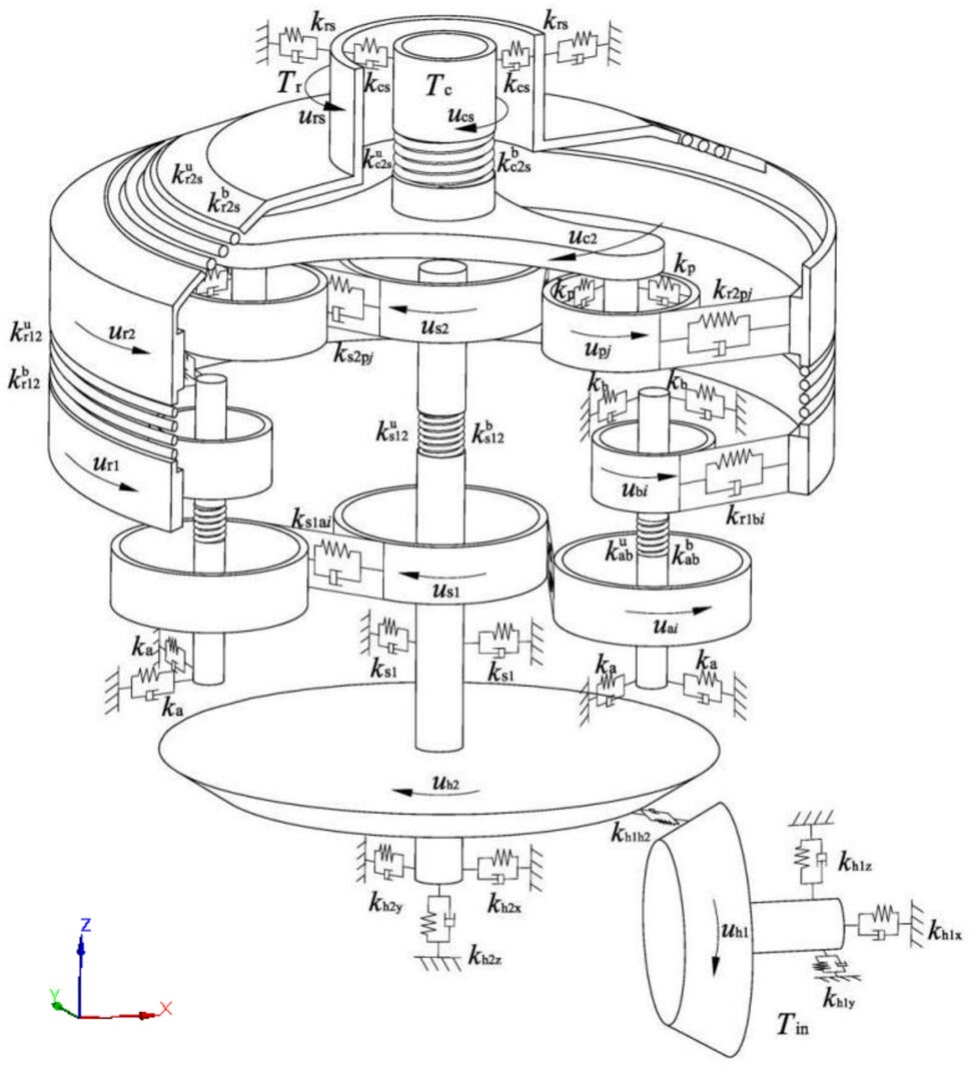
Translational-torsional dynamic model of the transmission system.

Without considering nonlinearities such as clearance, the dynamic model of the transmission system of the gearbox can be obtained by using the lumped parameter method, as shown in Fig. 3. The origin of the overall coordinate system of the transmission system is located at the intersection of the axes of the bevel gear pair. The positive direction of the X-axis of the coordinate system points to the input shaft, and the positive direction of the Z-axis points to the output shaft.

The dynamic model assumes that the size, mass, and moment of inertia of all planets in each planetary set are equal, the bearings of each planet have the same support stiffness and the stiffness in all directions is also equal.

The parameters in the model are defined as follows: $k_{m}$ is the mesh stiffness of gear pair *m*; $k_{h}$(*h* = *s*_1_, *r*_*s*_, *c*_*s*_, *a*, *b*, *p*) is the radial support stiffness of member *h*; $k_{{{\text{h1x}}}}$($k_{{{\text{h2x}}}}$), $k_{{{\text{h1y}}}}$($k_{{{\text{h2y}}}}$) and $k_{{{\text{h1z}}}}$($k_{{{\text{h2z}}}}$) are the three-way support stiffness of bevel gear *h*_1_(*h*_2_); and $k_{AB}^{{\text{b}}}$ and $k_{AB}^{{\text{u}}}$ are the radial coupling stiffness and torsional stiffness between members *A* and *B*, respectively. The definition of the damping symbol *c* is similar.

The vibration displacement vector of the system can be written as:

$$ \begin{aligned} {\varvec{q}} & { = }\left\{ {x_{{{\text{s}}1}} , y_{{{\text{s1}}}} , u_{{{\text{s1}}}} , x_{{{\text{r1}}}} , y_{{{\text{r1}}}} , u_{{{\text{r1}}}} , \zeta_{{{\text{a}}i}} , \eta_{{{\text{a}}i}} , u_{{{\text{a}}i}} , \zeta_{{{\text{b}}i}} , \eta_{{{\text{b}}i}} , u_{{{\text{b}}i}} , x_{{{\text{s}}2}} , } \right.y_{{{\text{s2}}}} , u_{{{\text{s2}}}} , x_{{{\text{r2}}}} {,} y_{{{\text{r2}}}} , u_{{{\text{r2}}}} , x_{{{\text{c2}}}} {, } \\ & \;\;\left. {y_{{{\text{c2}}}} , u_{{{\text{c2}}}} , \zeta_{{{\text{p}}j}} , \eta_{{{\text{p}}j}} , u_{{{\text{p}}j}} , x_{{{\text{cs}}}} , y_{{{\text{cs}}}} , u_{{{\text{cs}}}} , x_{{{\text{rs}}}} , y_{{{\text{rs}}}} , u_{{{\text{rs}}}} {,} x_{{{\text{h1}}}} , y_{{{\text{h1}}}} , z_{{{\text{h1}}}} , u_{{{\text{h1}}}} , x_{{{\text{h2}}}} , y_{{{\text{h2}}}} , z_{{{\text{h2}}}} , u_{{{\text{h2}}}} } \right\}^{{\text{T}}} \\ \end{aligned} $$

The vector includes *L* = 29 + 3(2*M* + *N*) degrees of freedom, where *x*, *y*, and *z* are the vibration displacements in the translational direction of the bevel gear, center gear, carrier and output shaft. $\zeta$ and $\eta$ are the vibration displacements of the planet along the radial and tangential directions of the carrier, respectively, and *u* is the torsional vibration line displacement of the component.

In the meshing cycle of a gear pair, the difference in mesh stiffness between single-tooth meshing and double-tooth meshing will cause large vibration acceleration of the gear system, which is an important excitation source of vibration of the gear system. The change in the mesh stiffness in the single-tooth meshing area or the double-tooth meshing area is small, which has little effect on the vibration response of the gear system^22^. Therefore, the time-varying mesh stiffness of gear pair *m* considering the mesh phase can be expanded into a Fourier series expression^23^:

4 $$ k_{m} \left( t \right) = k_{m}^{{\text{a}}} + 2k_{m}^{v} \mathop \sum \limits_{l = 1}^{\infty } \left( {a_{m}^{\left( l \right)} \cos l\omega_{m} t + b_{m}^{\left( l \right)} \sin l\omega_{m} t} \right) $$

where

$$ a_{m}^{\left( l \right)} = \frac{2}{\pi l}\cos \left[ {\pi l(2\gamma_{m} + \in_{m} )} \right]\sin \left( {\pi l \in_{m} } \right),\;b_{m}^{\left( l \right)} = \frac{2}{\pi l}\sin \left[ {\pi l(2\gamma_{m} + \in_{m} )} \right]\sin \left( {\pi l \in_{m} } \right) $$

$$ k_{m}^{{\text{a}}} = \left( { \in_{m} - 1} \right)k_{m}^{\max } + \left( {2 - \in_{m} } \right)k_{m}^{\min } ,\;\;2k_{m}^{v} = k_{m}^{\max } - k_{m}^{\min } $$

where $k_{m}^{{\text{a}}}$ and $2k_{m}^{v}$ are the mean value and fluctuation value of the mesh stiffness of gear pair *m,* respectively, and $\gamma_{m}$ is the mesh phase difference.

The mesh damping of gear pair *m* is:

5 $$ c_{m} = 2\zeta_{m} \sqrt {\frac{{I_{m1} \cdot I_{m2} }}{{I_{m1} r_{{{\text{bm}}2}}^{2} + I_{m2} r_{{{\text{bm}}1}}^{2} }}k_{m}^{{\text{a}}} } $$

where $\zeta_{{\text{m}}}$ is the mesh damping ratio of gear pair *m*, generally taken as 0.03–0.17; $I_{m1}$ and $I_{m2}$ are the moments of inertia of the driving and driven gears, respectively; and $r_{{{\text{bm1}}}}$ and $r_{{{\text{bm2}}}}$ are the base circle radii of the driving and driven gears, respectively.

### Relative displacement

Compression is defined as positive, and the relative displacement of the sun gear and planet along the meshing line is:

6 $$ \delta_{{{\text{s1a}}i}} = x_{{{\text{s1}}}} \sin \psi_{{{\text{s1a}}i}} - y_{{{\text{s1}}}} \cos \psi_{{{\text{s1a}}i}} - \zeta_{{a{\text{i}}}} \sin \alpha_{{{\text{s1}}}}^{\prime } + \eta_{{a{\text{i}}}} \cos \alpha_{{{\text{s}}1}}^{\prime } + u_{{{\text{s}}1}} - u_{{{\text{a}}i}} - e_{{{\text{s1a}}i}} $$

7 $$ \delta_{{{\text{s2p}}j}} = x_{{{\text{s2}}}} \sin \psi_{{{\text{s2p}}j}} - y_{{{\text{s}}2}} \cos \psi_{{{\text{s2p}}j}} - \zeta_{{{\text{p}}j}} \sin \alpha_{{{\text{s}}2}}^{\prime } + \eta_{{{\text{p}}j}} \cos \alpha_{{{\text{s}}2}}^{\prime } + u_{{{\text{s2}}}} - u_{{{\text{p}}j}} - e_{{{\text{s2p}}j}} $$

Similarly, the relative displacement between the ring gear and the planet is defined as:

8 $$ \delta_{{{\text{r1b}}i}} = \zeta_{{{\text{b}}i}} \sin \alpha_{{{\text{r}}1}}^{\prime } + \eta_{{{\text{b}}i}} \cos \alpha_{{{\text{r}}1}}^{\prime } - x_{{{\text{r}}1}} \sin \psi_{{{\text{r1b}}i}} - y_{{{\text{r}}1}} \cos \psi_{{{\text{r1b}}i}} + u_{{{\text{b}}i}} - u_{{{\text{r}}1}} - e_{{{\text{r1b}}i}} $$

9 $$ \delta_{{{\text{r2p}}j}} = \zeta_{{{\text{p}}j}} \sin \alpha_{{{\text{r}}2}}^{\prime } + \eta_{{{\text{p}}j}} \cos \alpha_{{{\text{r}}2}}^{\prime } - x_{{{\text{r}}2}} \sin \psi_{{{\text{r2p}}j}} - y_{{{\text{r}}2}} \cos \psi_{{{\text{r2p}}j}} + u_{{{\text{p}}j}} - u_{{{\text{r}}2}} - e_{{{\text{r2p}}j}} $$

where

10 $$ \begin{gathered} \psi_{{{\text{s1a}}i}} = \psi_{{{\text{a}}i}} + \alpha_{{{\text{s}}1}}^{\prime } ,\;\,\psi_{{{\text{r1b}}i}} = \alpha_{{{\text{r}}1}}^{\prime } - \psi_{{{\text{b}}i}} \hfill \\ \psi_{{{\text{s2p}}j}} = \psi_{{{\text{p}}j}} + \alpha_{{{\text{s}}2}}^{\prime } ,\;\psi_{{{\text{r2p}}j}} = \alpha_{{{\text{r}}2}}^{\prime } - \psi_{{{\text{p}}j}} \hfill \\ \end{gathered} $$

$\alpha_{{{\text{r1}}}}^{\prime }$ and $\alpha_{{{\text{s1}}}}^{\prime }$ are the working pressure angles of the internal and external meshing pair of the encased stage, respectively; $\alpha_{{{\text{r2}}}}^{\prime }$ and $\alpha_{{{\text{s2}}}}^{\prime }$ are the angles of the differential stage; and $\psi_{{{\text{a}}i}}$, $\psi_{{{\text{b}}i}}$ and $\psi_{{{\text{p}}j}}$ are the position angles of planet p*j*, which can be expressed as:

11 $$ \psi_{{{\text{a}}i}} = \psi_{{{\text{b}}i}} = 2\pi (i - 1)/M + \psi_{{{\text{a}}1}} ,\;\;\psi_{{{\text{p}}j}} = 2\pi (j - 1)/N + \psi_{{{\text{p1}}}} + \omega_{{{\text{c2}}}} t $$

The relative displacements of planet p_*j*_ and carrier *c*_2_ in the radial and tangential directions are as follows:

12 $$ \delta_{{{\text{c2p}}j}}^{\zeta } = \zeta_{{{\text{p}}j}} - x_{{{\text{c2}}}} \cos \psi_{{{\text{p}}j}} - y_{{{\text{c2}}}} \sin \psi_{{{\text{p}}j}} $$

13 $$ \delta_{{{\text{c2p}}j}}^{\eta } = \eta_{{{\text{p}}j}} + x_{{{\text{c2}}}} \sin \psi_{{{\text{p}}j}} - y_{{{\text{c2}}}} \cos \psi_{{{\text{p}}j}} + u_{{{\text{c2}}}} $$

The relative displacement of the bevel gear pair is:

14 $$ \delta_{{{\text{h1h2}}}} = c_{x} \left( {x_{{{\text{h1}}}} - x_{{{\text{h2}}}} } \right) + c_{y} \left( {y_{{{\text{h1}}}} - y_{{{\text{h2}}}} - u_{{{\text{h1}}}} + u_{{{\text{h2}}}} } \right) + c_{z} \left( {z_{{{\text{h1}}}} - z_{{{\text{h2}}}} } \right) - e_{{{\text{h1h2}}}} $$

where

$$ c_{x} = - \left( {\sin \delta_{{{\text{h2}}}} \cdot \sin \beta_{{{\text{h2}}}} \cdot \cos \alpha_{{{\text{h2}}}} + \cos \delta_{{{\text{h2}}}} \cdot \sin \alpha_{{{\text{h2}}}} } \right) $$

$$ c_{y} = - \cos \beta_{{{\text{h2}}}} \cdot \cos \alpha_{{{\text{h2}}}} $$

$$ c_{z} = \sin \delta_{{{\text{h2}}}} \cdot \sin \alpha_{{{\text{h2}}}} - \cos \delta_{{{\text{h2}}}} \cdot \sin \beta_{{{\text{h2}}}} \cdot \cos \alpha_{{{\text{h2}}}} $$

In the formula, $\alpha_{{{\text{h2}}}}$, $\delta_{{{\text{h2}}}}$, and $\beta_{{{\text{h2}}}}$ are the normal pressure angle, pitch cone angle, and helix angle at the midpoint of the tooth width of bevel gear *h*_2_, respectively. $e_{m}$ is the comprehensive error of gear pair *m* along the meshing line.

### Dynamic differential equations

Based on the translational-torsional dynamics model, the differential equations of the transmission system are derived as follows:

1. Dynamic equations of encased stage sun gear *s*_1_
  15 $$ \begin{aligned} m_{{{\text{s1}}}} \ddot{x}_{{{\text{s1}}}} & + \mathop \sum \limits_{i = 1}^{M} \left( {k_{{{\text{s1a}}i}} \delta_{{{\text{s1a}}i}} + c_{{{\text{s1a}}i}} \dot{\delta }_{{{\text{s1a}}i}} } \right)\sin \psi_{{{\text{s1a}}i}} + k_{{{\text{s1}}}} x_{{{\text{s1}}}} + c_{{{\text{s1}}}} \dot{x}_{{{\text{s1}}}} + k_{{{\text{s12}}}}^{{\text{b}}} (x_{{{\text{s1}}}} - x_{{{\text{s2}}}} ) \\ & \;\; + c_{{{\text{s12}}}}^{{\text{b}}} (\dot{x}_{{{\text{s1}}}} - \dot{x}_{{{\text{s2}}}} ) + k_{{{\text{h2s1}}}}^{{\text{b}}} (x_{{{\text{s1}}}} - x_{{{\text{h2}}}} ) + c_{{{\text{h2s1}}}}^{{\text{b}}} (\dot{x}_{{{\text{s1}}}} - \dot{x}_{{{\text{h2}}}} ) = 0 \\ \end{aligned} $$
  16 $$ \begin{aligned} m_{{{\text{s1}}}} \ddot{y}_{{{\text{s1}}}} & - \mathop \sum \limits_{i = 1}^{M} \left( {k_{{{\text{s1a}}i}} \delta_{{{\text{s1a}}i}} + c_{{{\text{s1a}}i}} \dot{\delta }_{{{\text{s1a}}i}} } \right)\cos \psi_{{{\text{s1a}}i}} + k_{{{\text{s1}}}} y_{{{\text{s1}}}} + c_{{{\text{s1}}}} \dot{y}_{{{\text{s1}}}} + k_{{{\text{s12}}}}^{{\text{b}}} {(}y_{{{\text{s1}}}} - y_{{{\text{s2}}}} ) \\ & \;\; + c_{{{\text{s12}}}}^{{\text{b}}} (\dot{y}_{{{\text{s1}}}} - \dot{y}_{{{\text{s2}}}} ) + k_{{{\text{h2s1}}}}^{{\text{b}}} (y_{{{\text{s1}}}} - y_{{{\text{h2}}}} ) + c_{{{\text{h2s1}}}}^{{\text{b}}} (\dot{y}_{{{\text{s1}}}} - \dot{y}_{{{\text{h2}}}} ) = 0 \\ \end{aligned} $$
  17 $$ \begin{aligned} \frac{{I_{{{\text{s1}}}} }}{{r_{{{\text{bs1}}}}^{{2}} }}\ddot{u}_{{{\text{s1}}}} & + \mathop \sum \limits_{i = 1}^{M} \left( {k_{{{\text{s1a}}i}} \delta_{{{\text{s1a}}i}} + c_{{{\text{s1a}}i}} \dot{\delta }_{{{\text{s1a}}i}} } \right) + \frac{{k_{{{\text{s12}}}}^{{\text{u}}} }}{{r_{{{\text{bs1}}}} }}\left( {\frac{{u_{{{\text{s1}}}} }}{{r_{{{\text{bs1}}}} }} - \frac{{u_{{{\text{s2}}}} }}{{r_{{{\text{bs2}}}} }}} \right) + \frac{{c_{{{\text{s12}}}}^{{\text{u}}} }}{{r_{{{\text{bs1}}}} }}\left( {\frac{{\dot{u}_{{{\text{s1}}}} }}{{r_{{{\text{bs1}}}} }} - \frac{{\dot{u}_{{{\text{s2}}}} }}{{r_{{{\text{bs2}}}} }}} \right) \\ & \;\; + \frac{{k_{{{\text{h2s1}}}}^{{\text{u}}} }}{{r_{{{\text{bs1}}}} }}\left( {\frac{{u_{{{\text{s1}}}} }}{{r_{{{\text{bs1}}}} }} - \frac{{u_{{{\text{h2}}}} }}{{r_{{{\text{bh2}}}} }}} \right) + \frac{{c_{{{\text{h2s1}}}}^{{\text{u}}} }}{{r_{{{\text{bs1}}}} }}\left( {\frac{{\dot{u}_{{{\text{s1}}}} }}{{r_{{{\text{bs1}}}} }} - \frac{{\dot{u}_{{{\text{h2}}}} }}{{r_{{{\text{bh2}}}} }}} \right) = 0 \\ \end{aligned} $$
2. Dynamic equations of encased stage ring gear *r*_1_
  18 $$ m_{{{\text{r1}}}} \ddot{x}_{{{\text{r1}}}} - \mathop \sum \limits_{i = 1}^{M} \left( {k_{{{\text{r1bi}}}} \delta_{{{\text{r1b}}i}} + c_{{{\text{r1b}}i}} \dot{\delta }_{{{\text{r1b}}i}} } \right)\sin \psi_{{{\text{r1b}}i}} + k_{{{\text{r12}}}}^{{\text{b}}} \left( {x_{{{\text{r1}}}} - x_{{{\text{r2}}}} } \right) + c_{{{\text{r12}}}}^{{\text{b}}} \left( {\dot{x}_{{{\text{r1}}}} - \dot{x}_{{{\text{r2}}}} } \right) = 0 $$
  19 $$ m_{{{\text{r1}}}} \ddot{y}_{{{\text{r1}}}} - \mathop \sum \limits_{i = 1}^{M} \left( {k_{{{\text{r1b}}i}} \delta_{{{\text{r1b}}i}} + c_{{{\text{r1b}}i}} \dot{\delta }_{{{\text{r1b}}i}} } \right)\cos \psi_{{{\text{r1b}}i}} + k_{{{\text{r12}}}}^{{\text{b}}} \left( {y_{{{\text{r1}}}} - y_{{{\text{r2}}}} } \right) + c_{{{\text{r12}}}}^{{\text{b}}} \left( {\dot{y}_{{{\text{r1}}}} - \dot{y}_{{{\text{r2}}}} } \right) = 0 $$
  20 $$ \frac{{I_{{{\text{r1}}}} }}{{r_{{{\text{br1}}}}^{{2}} }}\ddot{u}_{{{\text{r1}}}} - \mathop \sum \limits_{i = 1}^{M} \left( {k_{{{\text{r1b}}i}} \delta_{{{\text{r1b}}i}} + c_{{{\text{r1b}}i}} \dot{\delta }_{{{\text{r1b}}i}} } \right) + \frac{{k_{{{\text{r12}}}}^{{\text{u}}} }}{{r_{{{\text{br1}}}} }}\left( {\frac{{u_{{{\text{r1}}}} }}{{r_{{{\text{br1}}}} }} - \frac{{u_{{{\text{r2}}}} }}{{r_{{{\text{br2}}}} }}} \right) + \frac{{c_{{{\text{r12}}}}^{{\text{u}}} }}{{r_{{{\text{br1}}}} }}\left( {\frac{{\dot{u}_{{{\text{r1}}}} }}{{r_{{{\text{br1}}}} }} - \frac{{\dot{u}_{{{\text{r2}}}} }}{{r_{{{\text{br2}}}} }}} \right) = 0 $$
3. Dynamic equations of encased stage stepped planet *a*_*i*_
  21 $$ m_{a} \ddot{\zeta }_{{a{\text{i}}}} - \left( {k_{{s1a{\text{i}}}} \delta_{{s1a{\text{i}}}} + c_{{s1a{\text{i}}}} \dot{\delta }_{{s1a{\text{i}}}} } \right)\sin \alpha_{s1}^{\prime } + k_{a} \zeta_{{a{\text{i}}}} + c_{a} \dot{\zeta }_{{a{\text{i}}}} + k_{ab}^{b} (\zeta_{{a{\text{i}}}} - \zeta_{{b{\text{i}}}} ) + c_{ab}^{b} (\dot{\zeta }_{{a{\text{i}}}} - \dot{\zeta }_{{b{\text{i}}}} ) = 0 $$
  22 $$ m_{{\text{a}}} \ddot{\eta }_{{{\text{a}}i}} + \left( {k_{{{\text{s1a}}i}} \delta_{{{\text{s1a}}i}} + c_{{{\text{s1a}}i}} \dot{\delta }_{{{\text{s1a}}i}} } \right)\cos \alpha_{{{\text{s1}}}}^{\prime } + k_{{\text{a}}} \eta_{{{\text{a}}i}} + c_{{\text{a}}} \dot{\eta }_{{{\text{a}}i}} + k_{{{\text{ab}}}}^{{\text{b}}} {(}\eta_{{{\text{a}}i}} - \eta_{{{\text{b}}i}} ) + c_{{{\text{ab}}}}^{{\text{b}}} (\dot{\eta }_{{{\text{a}}i}} - \dot{\eta }_{{{\text{b}}i}} ) = 0 $$
  23 $$ \frac{{I_{{\text{a}}} }}{{r_{{{\text{ba}}}}^{{2}} }}\ddot{u}_{{{\text{a}}i}} - k_{{{\text{s1a}}i}} \delta_{{{\text{s1a}}i}} - c_{{{\text{s1a}}i}} \dot{\delta }_{{{\text{s1a}}i}} + \frac{{k_{{{\text{ab}}}}^{{\text{u}}} }}{{r_{{{\text{ba}}}} }}\left( {\frac{{u_{{{\text{a}}i}} }}{{r_{{{\text{ba}}}} }} - \frac{{u_{{{\text{b}}i}} }}{{r_{{{\text{bb}}}} }}} \right) + \frac{{c_{{{\text{ab}}}}^{{\text{u}}} }}{{r_{{{\text{ba}}}} }}\left( {\frac{{\dot{u}_{{{\text{a}}i}} }}{{r_{{{\text{ba}}}} }} - \frac{{\dot{u}_{{{\text{b}}i}} }}{{r_{{{\text{bb}}}} }}} \right) = 0 $$
4. Dynamic equations of encased stage stepped planet *b*_*i*_
  24 $$ m_{{\text{b}}} \ddot{\zeta }_{{{\text{b}}i}} + \left( {k_{{{\text{r1b}}i}} \delta_{{{\text{r1b}}i}} + c_{{{\text{r1b}}i}} \dot{\delta }_{{{\text{r1b}}i}} } \right)\sin \alpha_{{{\text{r}}1}}^{\prime } + k_{{\text{b}}} \zeta_{{{\text{b}}i}} + c_{{\text{b}}} \dot{\zeta }_{{{\text{b}}i}} - k_{{{\text{ab}}}}^{{\text{b}}} \left( {\zeta_{{{\text{a}}i}} - \zeta_{{{\text{b}}i}} } \right) - c_{{{\text{ab}}}}^{{\text{b}}} \left( {\dot{\zeta }_{{{\text{a}}i}} - \dot{\zeta }_{{{\text{b}}i}} } \right) = 0 $$
  25 $$ m_{{\text{b}}} \ddot{\eta }_{{{\text{b}}i}} + \left( {k_{{{\text{r1b}}i}} \delta_{{{\text{r1b}}i}} + c_{{{\text{r1b}}i}} \dot{\delta }_{{{\text{r1b}}i}} } \right)\cos \alpha_{{{\text{r}}1}}^{\prime } + k_{{\text{b}}} \eta_{{{\text{b}}i}} + c_{{\text{b}}} \dot{\eta }_{{{\text{b}}i}} - k_{{{\text{ab}}}}^{{\text{b}}} (\eta_{{{\text{a}}i}} - \eta_{{{\text{b}}i}} ) - c_{{{\text{ab}}}}^{{\text{b}}} \left( {\dot{\eta }_{{{\text{a}}i}} - \dot{\eta }_{{{\text{b}}i}} } \right) = 0 $$
  26 $$ \frac{{I_{{\text{b}}} }}{{r_{{{\text{bb}}}}^{{2}} }}\ddot{u}_{{{\text{b}}i}} + k_{{{\text{r1b}}i}} \delta_{{{\text{r1b}}i}} + c_{{{\text{r1b}}i}} \dot{\delta }_{{{\text{r1b}}i}} - \frac{{k_{{{\text{ab}}}}^{{\text{u}}} }}{{r_{{{\text{bb}}}} }}\left( {\frac{{u_{{{\text{a}}i}} }}{{r_{{{\text{ba}}}} }} - \frac{{u_{{{\text{b}}i}} }}{{r_{{{\text{bb}}}} }}} \right) - \frac{{c_{{{\text{ab}}}}^{{\text{u}}} }}{{r_{{{\text{bb}}}} }}\left( {\frac{{\dot{u}_{{{\text{a}}i}} }}{{r_{{{\text{ba}}}} }} - \frac{{\dot{u}_{{{\text{b}}i}} }}{{r_{{{\text{bb}}}} }}} \right) = 0 $$
5. Dynamic equations of differential stage sun gear *s*_2_
  27 $$ m_{{{\text{s2}}}} \ddot{x}_{{{\text{s2}}}} + \mathop \sum \limits_{j = 1}^{N} \left( {k_{{{\text{s2p}}j}} \delta_{{{\text{s2p}}j}} + c_{{{\text{s2p}}j}} \dot{\delta }_{{{\text{s2p}}j}} } \right)\sin \psi_{{{\text{s2p}}j}} + k_{{{\text{s12}}}}^{{\text{b}}} (x_{{{\text{s2}}}} - x_{{{\text{s1}}}} ) + {\text{c}}_{{{\text{s12}}}}^{{\text{b}}} (\dot{x}_{{{\text{s2}}}} - \dot{x}_{s1} ) = 0 $$
  28 $$ m_{{{\text{s2}}}} \ddot{y}_{{{\text{s2}}}} - \mathop \sum \limits_{j = 1}^{N} \left( {k_{{{\text{s2p}}j}} \delta_{{{\text{s2p}}j}} + c_{{{\text{s2p}}j}} \dot{\delta }_{{{\text{s2p}}j}} } \right)\cos \psi_{{{\text{s2p}}j}} + k_{{{\text{s12}}}}^{{\text{b}}} (y_{{{\text{s2}}}} - y_{{{\text{s1}}}} ) + c_{{{\text{s12}}}}^{{\text{b}}} (\dot{y}_{{{\text{s2}}}} - \dot{y}_{{{\text{s1}}}} ){ = 0} $$
  29 $$ \frac{{I_{{{\text{s2}}}} }}{{r_{{{\text{bs2}}}}^{{2}} }}\ddot{u}_{{{\text{s2}}}} + \mathop \sum \limits_{j = 1}^{N} \left( {k_{{{\text{s2p}}j}} \delta_{{{\text{s2p}}j}} + c_{{{\text{s2p}}j}} \dot{\delta }_{{{\text{s2p}}j}} } \right) - \frac{{k_{{{\text{s12}}}}^{{\text{u}}} }}{{r_{{{\text{bs2}}}} }}\left( {\frac{{u_{{{\text{s1}}}} }}{{r_{{{\text{bs1}}}} }} - \frac{{u_{{{\text{s2}}}} }}{{r_{{{\text{bs2}}}} }}} \right) - \frac{{c_{{{\text{s12}}}}^{{\text{u}}} }}{{r_{{{\text{bs2}}}} }}\left( {\frac{{\dot{u}_{{{\text{s1}}}} }}{{r_{{{\text{bs1}}}} }} - \frac{{\dot{u}_{{{\text{s2}}}} }}{{r_{{{\text{bs2}}}} }}} \right) = 0 $$
6. Dynamic equations of differential stage ring gear *r*_2_
  30 $$ m_{{{\text{r2}}}} \ddot{x}_{{{\text{r2}}}} { - }\mathop \sum \limits_{j = 1}^{N} \left( {k_{{{\text{r2p}}j}} \delta_{{{\text{r2p}}j}} + c_{{{\text{r2p}}j}} \dot{\delta }_{{{\text{r2p}}j}} } \right)\sin \psi_{{{\text{r2p}}j}} + k_{{{\text{r12}}}}^{{\text{b}}} \left( {x_{{{\text{r2}}}} - x_{{{\text{r1}}}} } \right) + c_{{{\text{r12}}}}^{{\text{b}}} \left( {\dot{x}_{{{\text{r2}}}} - \dot{x}_{{{\text{r1}}}} } \right) + k_{{{\text{r2s}}}}^{{\text{b}}} (x_{{{\text{r2}}}} - x_{{{\text{rs}}}} ) + c_{{{\text{r2s}}}}^{{\text{b}}} (\dot{x}_{{{\text{r2}}}} - \dot{x}_{{{\text{rs}}}} ) = 0 $$
  31 $$ m_{{{\text{r2}}}} \ddot{y}_{{{\text{r2}}}} - \mathop \sum \limits_{j = 1}^{N} \left( {k_{{{\text{r2p}}j}} \delta_{{{\text{r2p}}j}} + c_{{{\text{r2p}}j}} \dot{\delta }_{{{\text{r2p}}j}} } \right)\cos \psi_{{{\text{r2p}}j}} { + }k_{{{\text{r12}}}}^{{\text{b}}} \left( {y_{{{\text{r2}}}} y - y_{{{\text{r1}}}} } \right) + c_{{{\text{r12}}}}^{{\text{b}}} \left( {\dot{y}_{{{\text{r2}}}} - \dot{y}_{{{\text{r1}}}} } \right) + k_{{{\text{r2s}}}}^{{\text{b}}} (y_{{{\text{r2}}}} - y_{{{\text{rs}}}} ) + c_{{{\text{r2s}}}}^{{\text{b}}} (\dot{y}_{{{\text{r2}}}} - \dot{y}_{{{\text{rs}}}} ) = 0 $$
  32 $$ \frac{{I_{{{\text{r2}}}} }}{{r_{{{\text{br2}}}}^{{2}} }}\ddot{u}_{{{\text{r2}}}} - \mathop \sum \limits_{j = 1}^{N} \left( {k_{{{\text{r2p}}j}} \delta_{{{\text{r2p}}j}} + c_{{{\text{r2p}}j}} \dot{\delta }_{{{\text{r2p}}j}} } \right) - \frac{{k_{{{\text{r12}}}}^{{\text{u}}} }}{{r_{{{\text{br2}}}} }}\left( {\frac{{u_{{{\text{r1}}}} }}{{r_{{{\text{br1}}}} }} - \frac{{u_{{{\text{r2}}}} }}{{r_{{{\text{br2}}}} }}} \right) - \frac{{c_{{{\text{r12}}}}^{{\text{u}}} }}{{r_{{{\text{br2}}}} }}\left( {\frac{{\dot{u}_{{{\text{r1}}}} }}{{r_{{{\text{br1}}}} }} - \frac{{\dot{u}_{{{\text{r2}}}} }}{{r_{{{\text{br2}}}} }}} \right) + \frac{{k_{{{\text{r2s}}}}^{{\text{u}}} }}{{r_{{{\text{br2}}}} }}\left( {\frac{{u_{{{\text{r2}}}} }}{{r_{{{\text{br2}}}} }} - \frac{{u_{{{\text{rs}}}} }}{{r_{{{\text{rs}}}} }}} \right) + \frac{{c_{{{\text{r2s}}}}^{{\text{u}}} }}{{r_{{{\text{br2}}}} }}\left( {\frac{{\dot{u}_{{{\text{r2}}}} }}{{r_{{{\text{br2}}}} }} - \frac{{\dot{u}_{rs} }}{{r_{{{\text{rs}}}} }}} \right) = 0 $$
7. Dynamic equations of differential stage carrier *c*_2_
  33 $$ m_{{{\text{c2}}}} \ddot{x}_{{{\text{c2}}}} + k_{{{\text{c2s}}}}^{{\text{b}}} \left( {x_{{{\text{c2}}}} - x_{{{\text{cs}}}} } \right) + c_{{{\text{c2s}}}}^{{\text{b}}} \left( {\dot{x}_{{{\text{c2}}}} - \dot{x}_{{{\text{cs}}}} } \right) - \mathop \sum \limits_{j = 1}^{N} \left( {k_{{\text{p}}} \delta_{{{\text{c2p}}j}}^{\zeta } + c_{{\text{p}}} \dot{\delta }_{{{\text{c2p}}j}}^{\zeta } } \right)\cos \psi_{{{\text{p}}j}} + \mathop \sum \limits_{j = 1}^{N} \left( {k_{{\text{p}}} \delta_{{{\text{c2p}}j}}^{\eta } + c_{{\text{p}}} \dot{\delta }_{{{\text{c2p}}j}}^{\eta } } \right)\sin \psi_{{{\text{p}}j}} = 0 $$
  34 $$ m_{c2} \ddot{y}_{c2} + k_{{{\text{c2s}}}}^{{\text{b}}} \left( {y_{{{\text{c2}}}} - y_{{{\text{cs}}}} } \right) + c_{{{\text{c2s}}}}^{{\text{b}}} \left( {\dot{y}_{{{\text{c2}}}} - \dot{y}_{{{\text{cs}}}} } \right) - \mathop \sum \limits_{j = 1}^{N} \left( {k_{{\text{p}}} \delta_{{{\text{c2p}}j}}^{\zeta } + c_{{\text{p}}} \dot{\delta }_{{{\text{c2p}}j}}^{\zeta } } \right)\sin \psi_{{{\text{p}}j}} - \mathop \sum \limits_{j = 1}^{N} \left( {k_{{\text{p}}} \delta_{{{\text{c2p}}j}}^{\eta } + c_{{\text{p}}} \dot{\delta }_{{{\text{c2p}}j}}^{\eta } } \right)\cos \psi_{{{\text{p}}j}} = 0 $$
  35 $$ \frac{{I_{{{\text{c2}}}} }}{{r_{{{\text{c2}}}}^{{2}} }}\ddot{u}_{c2} + \frac{{k_{{{\text{c2s}}}}^{{\text{u}}} }}{{r_{{{\text{c2}}}} }}\left( {\frac{{u_{{{\text{c2}}}} }}{{r_{{{\text{c2}}}} }} - \frac{{u_{{{\text{cs}}}} }}{{r_{{{\text{cs}}}} }}} \right) + \frac{{c_{{{\text{c2s}}}}^{{\text{u}}} }}{{r_{{{\text{c2}}}} }}\left( {\frac{{\dot{u}_{{{\text{c2}}}} }}{{r_{{{\text{c2}}}} }} - \frac{{\dot{u}_{{{\text{cs}}}} }}{{r_{{{\text{cs}}}} }}} \right) + \mathop \sum \limits_{j = 1}^{N} \left( {k_{{\text{p}}} \delta_{{{\text{c2p}}j}}^{\eta } + c_{{\text{p}}} \dot{\delta }_{{{\text{c2p}}j}}^{\eta } } \right) = 0 $$
8. Dynamic equations of differential stage planet *p*_*j*_
  36 $$ m_{{\text{p}}} \left( {\ddot{\zeta }_{{{\text{p}}j}} + 2\omega_{{{\text{c2}}}} \dot{\eta }_{{{\text{p}}j}} - \omega_{{{\text{c2}}}}^{{2}} \zeta_{{{\text{p}}j}} - \omega_{{{\text{c2}}}}^{{2}} r_{{{\text{c2}}}} } \right) + k_{{\text{p}}} \delta_{{{\text{c2p}}j}}^{\zeta } + c_{{\text{p}}} \dot{\delta }_{{{\text{c2p}}j}}^{\zeta } - \left( {k_{{{\text{s2p}}j}} \delta_{{{\text{s2p}}j}} + c_{{{\text{s2p}}j}} \dot{\delta }_{{{\text{s2p}}j}} } \right)\sin \alpha_{{{\text{s2}}}}^{\prime } + \left( {k_{{{\text{r2p}}j}} \delta_{{{\text{r2p}}j}} + c_{{{\text{r2p}}j}} \dot{\delta }_{{{\text{r2p}}j}} } \right)\sin \alpha_{{{\text{r}}2}}^{\prime } = 0 $$
  37 $$ m_{{\text{p}}} \left( {\ddot{\eta }_{{{\text{p}}j}} - 2\omega_{{{\text{c2}}}} \dot{\zeta }_{{{\text{p}}j}} - \omega_{{{\text{c2}}}}^{{2}} \eta_{{{\text{p}}j}} } \right) + k_{{\text{p}}} \delta_{{{\text{c2p}}j}}^{\eta } + c_{{\text{p}}} \dot{\delta }_{{{\text{c2p}}j}}^{\eta } + \left( {k_{{{\text{s2p}}j}} \delta_{{{\text{s2p}}j}} + c_{{{\text{s2p}}j}} \dot{\delta }_{{{\text{s2p}}j}} } \right)\cos \alpha_{{{\text{s}}2}}^{\prime } + \left( {k_{{{\text{r2p}}j}} \delta_{{{\text{r2p}}j}} + c_{{{\text{r2p}}j}} \dot{\delta }_{{{\text{r2p}}j}} } \right)\cos \alpha_{{{\text{r}}2}}^{\prime } = 0 $$
  38 $$ \frac{{I_{{\text{p}}} }}{{r_{{{\text{bp}}}}^{{2}} }}\ddot{u}_{{{\text{p}}j}} - k_{{{\text{s2p}}j}} \delta_{{{\text{s2p}}j}} - c_{{{\text{s2p}}j}} \dot{\delta }_{{{\text{s2p}}j}} + k_{{{\text{r2p}}j}} \delta_{{{\text{r2p}}j}} + c_{{{\text{r2p}}j}} \dot{\delta }_{{{\text{r2p}}j}} = 0 $$
9. Dynamic equations of the inner output shaft *c*_*s*_
  39 $$ m_{{{\text{cs}}}} \ddot{x}_{{{\text{cs}}}} + k_{{{\text{c2s}}}}^{{\text{b}}} \left( {x_{{{\text{cs}}}} - x_{{{\text{c2}}}} } \right) + c_{{{\text{c2s}}}}^{{\text{b}}} \left( {\dot{x}_{{{\text{cs}}}} - \dot{x}_{{{\text{c2}}}} } \right) + k_{{{\text{cs}}}} (x_{{{\text{cs}}}} - x_{{{\text{rs}}}} ) + c_{{{\text{cs}}}} (\dot{x}_{{{\text{cs}}}} - \dot{x}_{{{\text{rs}}}} ) = 0 $$
  40 $$ m_{{{\text{cs}}}} \ddot{y}_{{{\text{cs}}}} + k_{{{\text{c2s}}}}^{{\text{b}}} \left( {y_{{{\text{cs}}}} - y_{{{\text{c2}}}} } \right) + c_{{{\text{c2s}}}}^{{\text{b}}} \left( {\dot{y}_{{{\text{cs}}}} - \dot{y}_{{{\text{c2}}}} } \right) + k_{{{\text{cs}}}} (y_{{{\text{cs}}}} - y_{{{\text{rs}}}} ) + c_{{{\text{cs}}}} (\dot{y}_{{{\text{cs}}}} - \dot{y}_{{{\text{rs}}}} ) = 0 $$
  41 $$ \frac{{I_{{{\text{cs}}}} }}{{r_{{{\text{cs}}}}^{{2}} }}\ddot{u}_{{{\text{cs}}}} - \frac{{k_{{{\text{c2s}}}}^{{\text{u}}} }}{{r_{{{\text{cs}}}} }}\left( {\frac{{u_{{{\text{c2}}}} }}{{r_{{{\text{c2}}}} }} - \frac{{u_{{{\text{cs}}}} }}{{r_{{{\text{cs}}}} }}} \right) - \frac{{c_{{{\text{c2s}}}}^{{\text{u}}} }}{{r_{{{\text{cs}}}} }}\left( {\frac{{\dot{u}_{{{\text{c2}}}} }}{{r_{{{\text{c}}2}} }} - \frac{{\dot{u}_{{{\text{cs}}}} }}{{r_{{{\text{cs}}}} }}} \right) = - \frac{{T_{{\text{c}}} \left( t \right)}}{{r_{{{\text{cs}}}} }} $$
10. Dynamic equations of outer output shaft *r*_*s*_
  42 $$ m_{{{\text{rs}}}} \ddot{x}_{{{\text{rs}}}} + k_{{{\text{r2s}}}}^{{\text{b}}} \left( {x_{{{\text{rs}}}} - x_{{{\text{r2}}}} } \right) + c_{{{\text{r2s}}}}^{{\text{b}}} \left( {\dot{x}_{{{\text{rs}}}} - \dot{x}_{{{\text{r2}}}} } \right) + k_{{{\text{cs}}}} \left( {x_{{{\text{rs}}}} - x_{{{\text{cs}}}} } \right) + c_{{{\text{cs}}}} \left( {\dot{x}_{{{\text{rs}}}} - \dot{x}_{{{\text{cs}}}} } \right) + k_{{{\text{rs}}}} x_{{{\text{rs}}}} + c_{{{\text{rs}}}} \dot{x}_{{{\text{rs}}}} = 0 $$
  43 $$ m_{{{\text{rs}}}} \ddot{y}_{{{\text{rs}}}} + k_{{{\text{r2s}}}}^{{\text{b}}} \left( {y_{{{\text{rs}}}} - y_{{{\text{r2}}}} } \right) + c_{{{\text{r2s}}}}^{{\text{b}}} \left( {\dot{y}_{{{\text{rs}}}} - \dot{y}_{{{\text{r2}}}} } \right) + k_{{{\text{cs}}}} \left( {y_{{{\text{rs}}}} - y_{{{\text{cs}}}} } \right) + c_{{{\text{cs}}}} \left( {\dot{y}_{{{\text{rs}}}} - \dot{y}_{{{\text{cs}}}} } \right) + k_{{{\text{rs}}}} y_{{{\text{rs}}}} + c_{{{\text{rs}}}} \dot{y}_{{{\text{rs}}}} = {0} $$
  44 $$ \frac{{I_{{{\text{rs}}}} }}{{r_{{{\text{rs}}}}^{{2}} }}\ddot{u}_{{{\text{rs}}}} - \frac{{k_{{{\text{r2s}}}}^{{\text{u}}} }}{{r_{{{\text{rs}}}} }}\left( {\frac{{u_{{{\text{r2}}}} }}{{r_{{{\text{br2}}}} }} - \frac{{u_{{{\text{rs}}}} }}{{r_{{{\text{rs}}}} }}} \right) - \frac{{c_{{{\text{r2s}}}}^{{\text{u}}} }}{{r_{{{\text{rs}}}} }}\left( {\frac{{\dot{u}_{{{\text{r2}}}} }}{{r_{{{\text{br2}}}} }} - \frac{{\dot{u}_{{{\text{rs}}}} }}{{r_{{{\text{rs}}}} }}} \right) = - \frac{{T_{{\text{r}}} \left( t \right)}}{{r_{{{\text{rs}}}} }} $$
11. Dynamic equations of bevel gear *h*_1_:
  45 $$ m_{{{\text{h1}}}} \ddot{x}_{{{\text{h1}}}} + c_{{{\text{h1}}x}} \dot{x}_{{{\text{h1}}}} + k_{{{\text{h1}}x}} x_{{{\text{h1}}}} + c_{x} \left( {k_{{{\text{h1h2}}}} \delta_{{{\text{h1h2}}}} + c_{{{\text{h1h2}}}} \dot{\delta }_{{{\text{h1h2}}}} } \right) = 0 $$
  46 $$ m_{{{\text{h1}}}} \ddot{y}_{{{\text{h1}}}} + c_{{{\text{h1}}y}} \dot{y}_{{{\text{h1}}}} + k_{{{\text{h1}}y}} y_{{{\text{h1}}}} + c_{y} \left( {k_{{{\text{h1h2}}}} \delta_{{{\text{h1h2}}}} + c_{{{\text{h1h2}}}} \dot{\delta }_{{{\text{h1h2}}}} } \right) = 0 $$
  47 $$ m_{{{\text{h1}}}} \ddot{z}_{{{\text{h1}}}} + c_{{{\text{h1}}z}} \dot{z}_{{{\text{h1}}}} + k_{{{\text{h}}1z}} z_{{{\text{h1}}}} + c_{z} \left( {k_{{{\text{h1h2}}}} \delta_{{{\text{h1h2}}}} + c_{{{\text{h1h2}}}} \dot{\delta }_{{{\text{h1h2}}}} } \right) = 0 $$
  48 $$ \frac{{I_{{{\text{h1}}}} }}{{r_{{{\text{bh1}}}}^{{2}} }}\ddot{u}_{{{\text{h1}}}} - c_{y} \left( {k_{{{\text{h1h2}}}} \delta_{{{\text{h1h2}}}} + c_{{{\text{h1h2}}}} \dot{\delta }_{{{\text{h1h2}}}} } \right) = \frac{{T_{{{\text{in}}}} \left( t \right)}}{{r_{{{\text{bh1}}}} }} $$
12. Dynamic equations of bevel gear *h*_2_:
  49 $$ m_{{{\text{h2}}}} \ddot{x}_{{{\text{h2}}}} + c_{{{\text{h2}}x}} \dot{x}_{{{\text{h2}}}} + k_{{{\text{h2}}x}} x_{{{\text{h2}}}} + k_{{{\text{h2s1}}}}^{{\text{b}}} (x_{{{\text{h2}}}} - x_{{{\text{s1}}}} ) + c_{{{\text{h2s1}}}}^{{\text{b}}} (\dot{x}_{{{\text{h2}}}} - \dot{x}_{{{\text{s1}}}} ) - {\text{c}}_{x} \left( {k_{{{\text{h1h2}}}} \delta_{{{\text{h1h2}}}} + c_{{{\text{h1h2}}}} \dot{\delta }_{{{\text{h1h2}}}} } \right) = 0 $$
  50 $$ m_{{{\text{h2}}}} \ddot{y}_{{{\text{h2}}}} + c_{{{\text{h2}}y}} \dot{y}_{{{\text{h2}}}} + k_{{{\text{h2}}y}} y_{{{\text{h2}}}} + k_{{{\text{h2s1}}}}^{{\text{b}}} (y_{{{\text{h2}}}} - y_{{{\text{s1}}}} ) + c_{{{\text{h2s1}}}}^{{\text{b}}} (\dot{y}_{{{\text{h2}}}} - \dot{y}_{{{\text{s1}}}} ) - c_{y} \left( {k_{{{\text{h1h2}}}} \delta_{{{\text{h1h2}}}} + c_{{{\text{h1h2}}}} \dot{\delta }_{{{\text{h1h2}}}} } \right) = 0 $$
  51 $$ m_{{{\text{h2}}}} \ddot{z}_{{{\text{h2}}}} + c_{{{\text{h2z}}}} \dot{z}_{{{\text{h2}}}} + k_{h2z} z_{h2} - c_{z} \left( {k_{{{\text{h1h2}}}} \delta_{{{\text{h1h2}}}} + c_{{{\text{h1h2}}}} \dot{\delta }_{{{\text{h1h2}}}} } \right) = 0 $$
  52 $$ \frac{{I_{{{\text{h2}}}} }}{{r_{{{\text{bh2}}}}^{{2}} }}\ddot{u}_{{{\text{h2}}}} + \frac{{k_{{{\text{h2s1}}}}^{{\text{u}}} }}{{r_{{{\text{bh2}}}} }}\left( {\frac{{u_{{{\text{h2}}}} }}{{r_{{{\text{bh2}}}} }} - \frac{{u_{{{\text{s1}}}} }}{{r_{{{\text{bs1}}}} }}} \right) + \frac{{c_{{{\text{h2s1}}}}^{{\text{u}}} }}{{r_{{{\text{bh2}}}} }}\left( {\frac{{\dot{u}_{{{\text{h2}}}} }}{{r_{{{\text{bh2}}}} }} - \frac{{\dot{u}_{{{\text{s1}}}} }}{{r_{{{\text{bs1}}}} }}} \right) + c_{y} \left( {k_{{{\text{h1h2}}}} \delta_{{{\text{h1h2}}}} + c_{{{\text{h1h2}}}} \dot{\delta }_{{{\text{h1h2}}}} } \right) = 0 $$

In the above equations, $I_{h}$ and $m_{h}$ (*h* = *s*_1_, *r*_1_, *a*, *b*, *s*_2_, *r*_2_, *c*_2_, *p*, *c*_*s*_, *r*_*s*_, *h*_1_, *h*_2_) are the moment of inertia and mass of the component, respectively, and ${\text{ r}}_{{{\text{bh}}}}$ is the radius of the base circle of the gear. $r_{h1}$ and $r_{h2}$ are the pitch circle radii of the bevel gear. $r_{cs}$ and $r_{rs}$ are the shaft diameters of the inner and outer shafts, respectively, and $r_{c2}$ is the distance from the center of the planet to the center of the carrier.

The expression in the matrix form of the dynamic equations is

53 $$ \user2{M\ddot{q} + C\dot{q}} + \omega_{{{\text{c2}}}} \user2{G\dot{q}} + \left( {{\varvec{K}}_{b} + {\varvec{K}}_{m} - \omega_{{{\text{c2}}}}^{{2}} {\varvec{K}}_{\omega } } \right)\user2{q = Q} $$

where ***M*** is the positive definite mass matrix, ***C*** is the damping matrix, ***G*** is the gyroscopic matrix, ${\varvec{K}}_{b}$ is the support stiffness, radial coupling stiffness and torsional stiffness matrix, ${\varvec{K}}_{m}$ is the mesh stiffness matrix, ${\varvec{K}}_{\omega }$ is the stiffness matrix caused by the gyroscopic effect, and ***Q*** is the vector of force and moment. The main parameters in the equations are shown in Table 2.

**Table 2:** Parameters of the gearbox.

Parameter	Values
Comprehensive meshing stiffness (N/m)	$k_{{{\text{s1a}}i}}^{a} = 5.80 \times 10^{8}$, $k_{{{\text{r1bi}}}}^{{\text{a}}} = 6.57 \times 10^{8}$, $k_{{{\text{s2pj}}}}^{{\text{a}}} = 9.91 \times 10^{8}$,$k_{{{\text{r2pj}}}}^{{\text{a}}} = 1.12 \times 10^{9}$
Mesh stiffness fluctuation value (N/m)	$k_{{{\text{s1ai}}}}^{{\text{v}}} = 1.50 \times 10^{8}$, $k_{{{\text{r1bi}}}}^{{\text{v}}} = 1.77 \times 10^{{8}}$, $k_{{{\text{s2pj}}}}^{{\text{v}}} = 2.49 \times 10^{8}$,$k_{{{\text{r2pj}}}}^{{\text{v}}} = 2.66 \times 10^{8}$
Bearing stiffness (N/m)	$k_{{{\text{s1}}}} = 3.5 \times 10^{8}$, $k_{{\text{a}}} = 2.6 \times 10^{{8}}$, $k_{{\text{b}}} = 3.5 \times 10^{8}$,$k_{{\text{p}}} = 5.2 \times 10^{8}$, $k_{{{\text{rs}}}} = 6.2 \times 10^{8}$,$k_{{{\text{cs}}}} = 2.9 \times 10^{8}$
Torsional coupling stiffness (N m/rad)	$k_{{{\text{s12}}}}^{{\text{u}}} = 2.4 \times 10^{6}$, $k_{{{\text{ab}}}}^{{\text{u}}} = 8.6 \times 10^{5}$, $k_{{{\text{r12}}}}^{{\text{u}}} = 5.7 \times 10^{8}$, $k_{{{\text{r2s}}}}^{{\text{u}}} = 3.8 \times 10^{7}$,$k_{{{\text{c2s}}}}^{{\text{u}}} = 3.2 \times 10^{6}$
Translation coupling stiffness (N/m)	$k_{{{\text{s12}}}}^{{\text{b}}} = 2.1 \times 10^{8}$, $k_{{{\text{ab}}}}^{{\text{b}}} = 1.8 \times 10^{9}$, $k_{{{\text{r12}}}}^{{\text{b}}} = 6.2 \times 10^{10}$, $k_{{{\text{r2s}}}}^{{\text{b}}} = 6.2 \times 10^{10}$,$k_{{{\text{c2s}}}}^{{\text{b}}} = 1.5 \times 10^{10}$
Working pressure angle ${(}^{ \circ } {)}$	$\alpha_{{{\text{s1}}}}^{\prime } = 2{2}{\text{.29}}$, $\alpha_{{{\text{r1}}}}^{\prime } = 19.22$, $\alpha_{{{\text{s2}}}}^{\prime } = \alpha_{{{\text{r2}}}}^{\prime } = {20}$*,*$\alpha_{{{\text{h1}}}}^{\prime } = {20}$
Contact ratio	$\in_{{s1a{\text{i}}}} = {1}{\text{.5972}}$, $\in_{{r1b{\text{i}}}} = {1}{\text{.5257}}$, $\in_{{s2p{\text{j}}}} = {1}{\text{.6575}}$, $\in_{{r2p{\text{j}}}} = {1}{\text{.7662}}$

## Dynamic model of the box

When the gearbox works under load, the flexibility of the box will have a greater impact on the force and deformation state of the transmission system^17,24^. Therefore, considering the coupling effect between the box and the transmission system is of great significance to accurately evaluate the dynamic characteristics of the main gearbox.

### Modal calculffation

**Figure 4:**
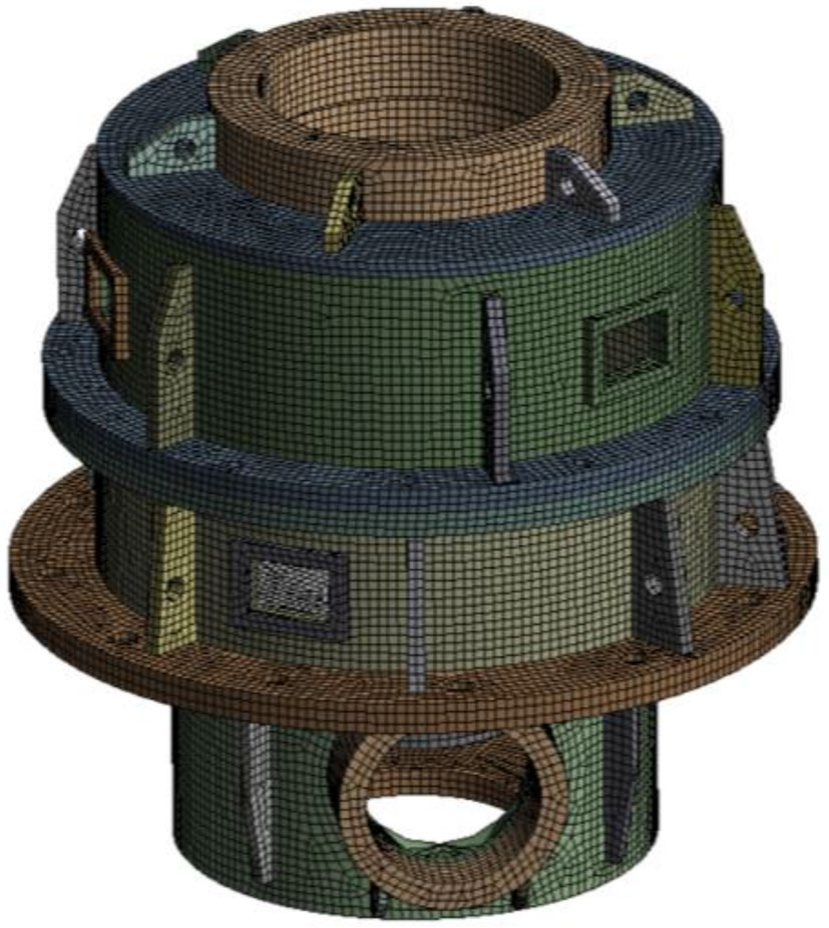
Finite element model of the box.

After geometric modeling and meshing, the finite element model of the box is obtained based on ANSYS software, as shown in Fig. 4. The material properties are set as follows: elastic modulus 206 GPa, Poisson's ratio 0.3, and density 7850 kg/m^3^.

**Figure 5:**
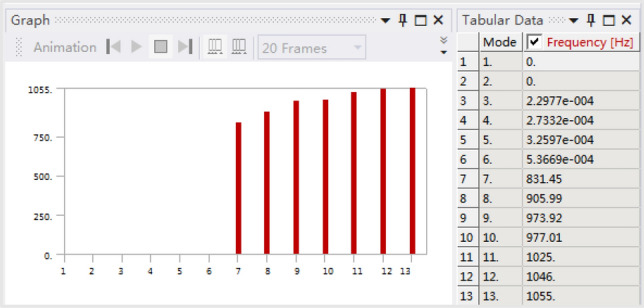
Natural frequencies of the box.

**Figure 6:**
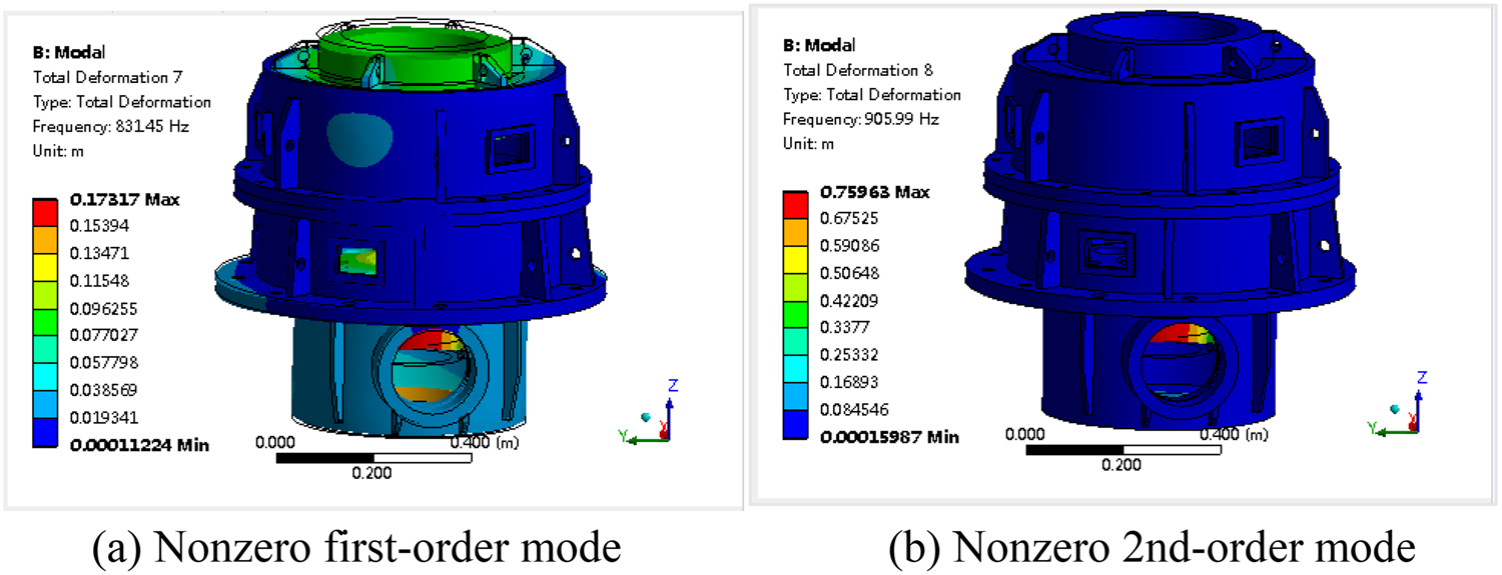
Vibration modes of the box.

The natural frequencies obtained by modal analysis of the box are shown in Fig. 5, and the corresponding partial vibration modes are shown in Fig. 6. Since the box is in an unconstrained state in the modal analysis, the first to sixth orders are rigid-body modes with zero frequency, so they will not be considered in the subsequent analysis. Among them, the nonzero first-order vibration mode is the breathing mode that increases or decreases along the axis direction, and the nonzero second-order vibration mode is the bending vibration of the web plate in the lower box.

### Modal testing

**Figure 7:**
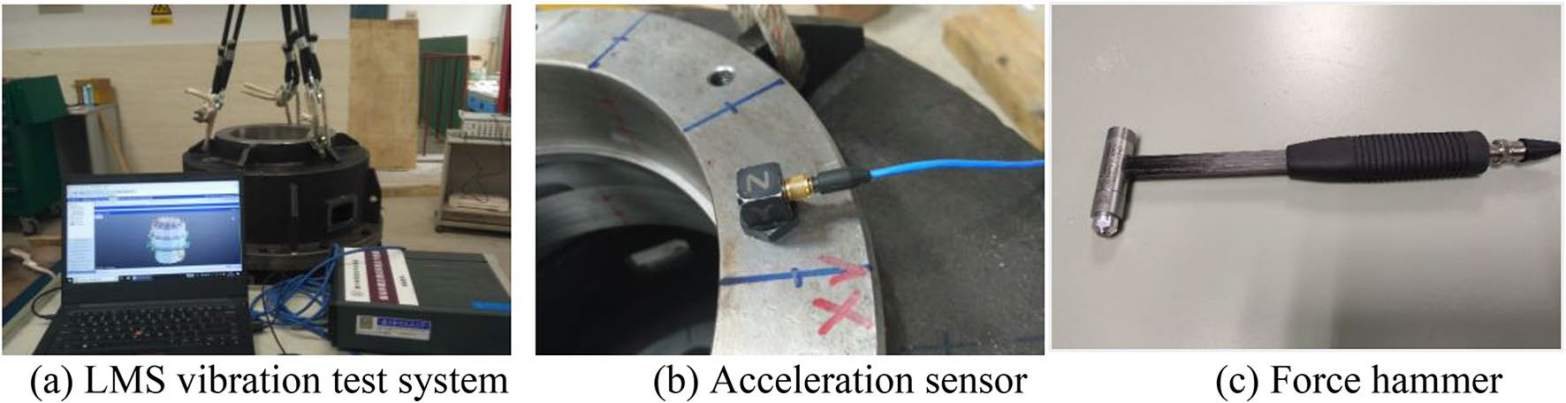
Modal testing equipment.

Modal testing is carried out by the impact hammer test method. The LMS Test. Lab vibration test system, the three-directional acceleration sensor, and the force hammer are used in the experiment, as shown in Fig. 7. The sensitivity of the acceleration sensor is 100 mV/g and that of the hammer force sensor is 0.218 mV/N.

**Figure 8:**
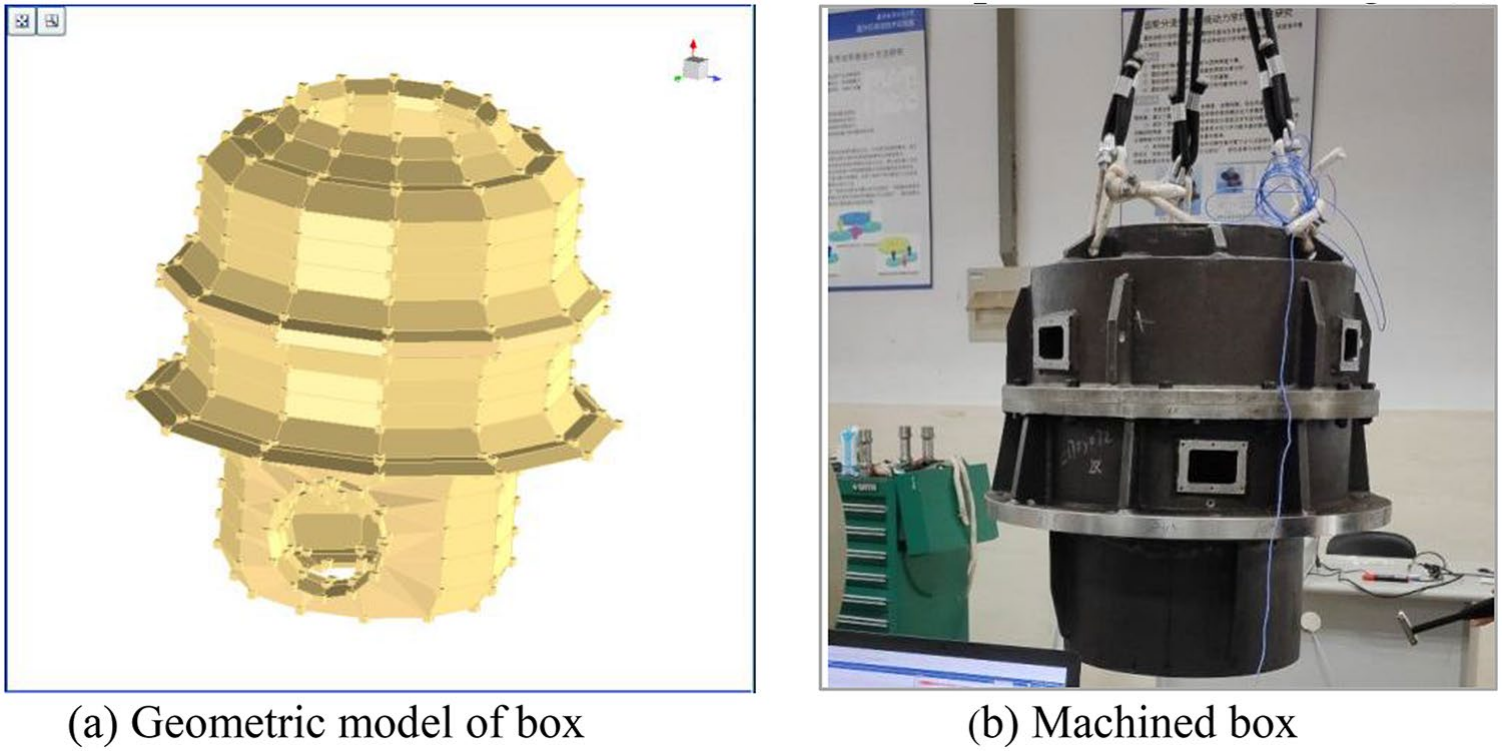
Geometric model and machined box.

In the geometry module, a geometric model of the box with a total of 488 points is established, as shown in Fig. 8a. Mark the position of the point corresponding to the geometric model on the box, stick the acceleration sensor on the box with a thin layer of beeswax, and hoist the box with an elastic rope, as shown in Fig. 8b.

**Figure 9:**
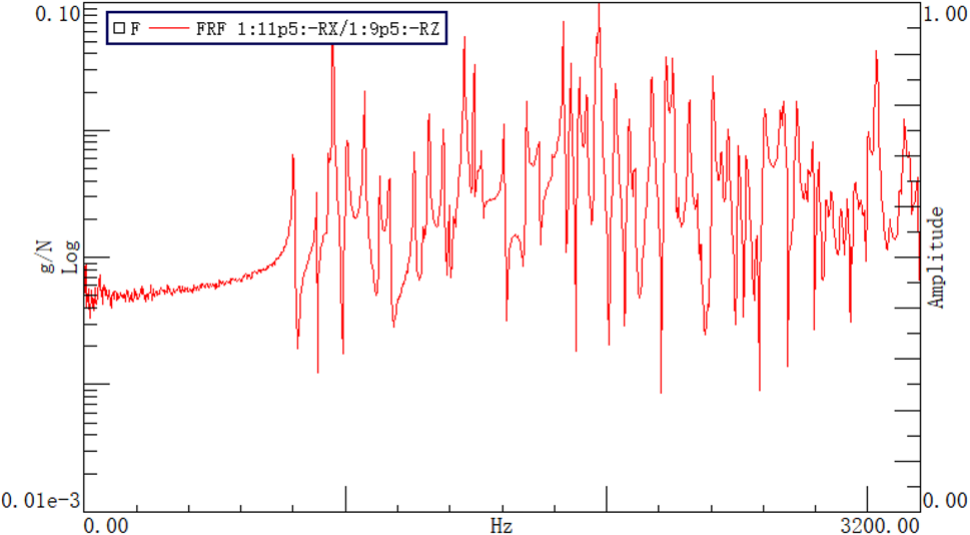
Natural frequencies of the box.

**Figure 10:**
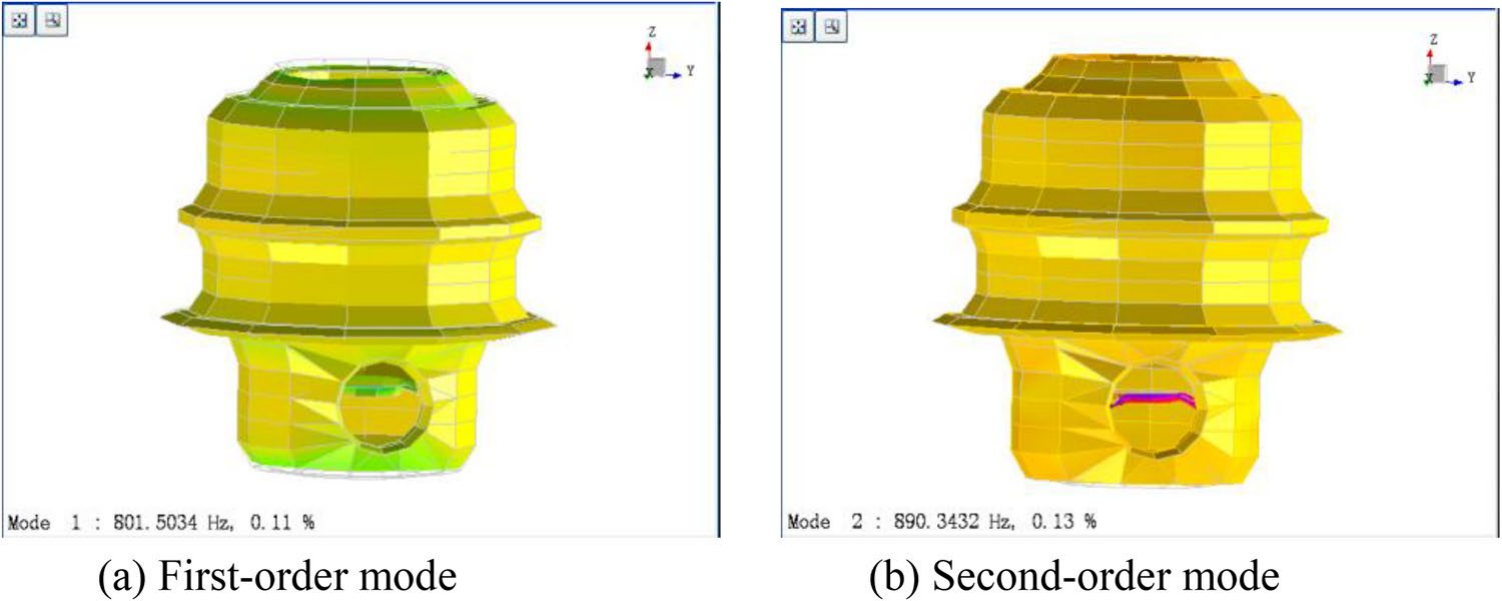
Vibration mode of the box.

Moving the force hammer and hammering all 488 points, the natural frequencies of the box are obtained by analyzing the cross frequency response functions, as shown in Fig. 9. The corresponding partial vibration modes are shown in Fig. 10, which are the same as the corresponding modal calculation results.

The results of the modal test and modal calculation are analyzed, and the frequencies corresponding to the same vibration mode of the first six orders are shown in Table 3. It can be seen from the table that the errors of these frequencies have no significant regularity and are all within 5%. The reason for the error is the difference in geometric dimensions and material properties between the machined box and the finite element model.

**Table 3:** Frequencies of modal calculation and testing.

Order	1	2	3	4	5	6
Calculation (Hz)	831.45	905.99	973.92	977.01	1046.00	1055.00
Test (Hz)	801.50	890.34	934.16	953.25	1006.37	1065.80
Error	3.74%	1.76%	4.26%	2.49%	3.94%	1.01%

### Model updating based on test data

Based on the modal test data, the finite element model of the box can be updated, and the accurate dynamic parameters of the box can be extracted to establish the rotor–stator coupling dynamic model.

1. Updated model of the box.

Considering the manufacturing process of the box, the main geometric and material parameters were selected as variable parameters: diameter, height, wall thickness, elastic modulus, and Poisson's ratio, and the parametric finite element calculation model for the box is established.

The minimum error between the calculation and experimental modal data is defined as the objective function. Considering that the effective mass of low-order frequencies accounts for the vast majority of the actual mass, only the first six nonzero natural frequencies are considered in the update process. Then,

54 $$ \min f(v_{j} ) = \left[ {\mathop \sum \limits_{i = 1}^{6} (f_{fi} - f_{ti} )^{2} } \right]^{1/2} ,\;\;\left( {v_{j}^{\min } \ll v_{j} \ll v_{j}^{\max } } \right) $$

In the formula, $f_{ti}$ is the frequency measured by the modal test, $f_{fi}$ is the frequency calculated by the finite element, and $v_{j}^{\max }$ and $v_{j}^{\min }$ are the upper and lower limits of the variable parameter $v_{j}$.

**Figure 11:**
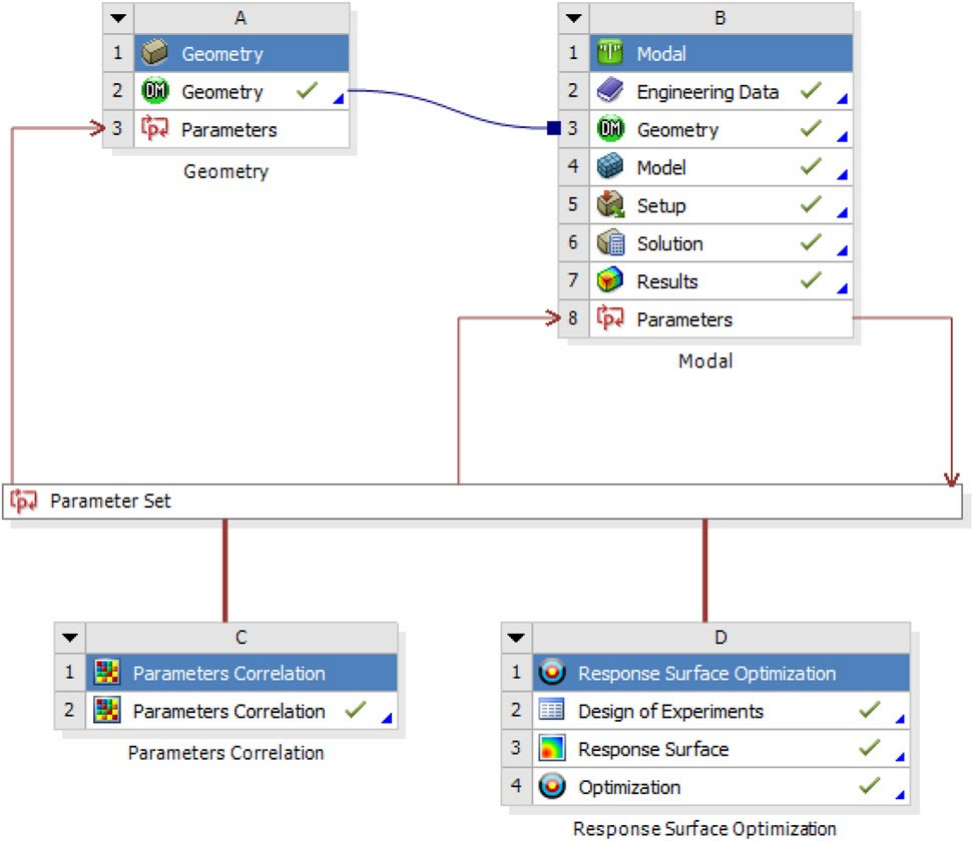
Optimization model based on the response surface.

The model updating is transformed into a constrained structural optimization problem, and an optimization model based on the response surface is established based on ANSYS software, as shown in Fig. 11. The error between the simulation frequency and the test frequency is defined as the output parameter, and the goal of minimizing the output parameter can be achieved by adjusting the variable parameters.

2. Updated results.

Correlation analysis of the parameters is carried out, and the response surface is calculated. After approximately 140 iterations, the target value remained stable and the updated parameters are obtained. Among them, the elastic modulus is 195 GPa, Poisson's ratio is 0.255, and density is 7830 kg/m^3^.

**Figure 12:**
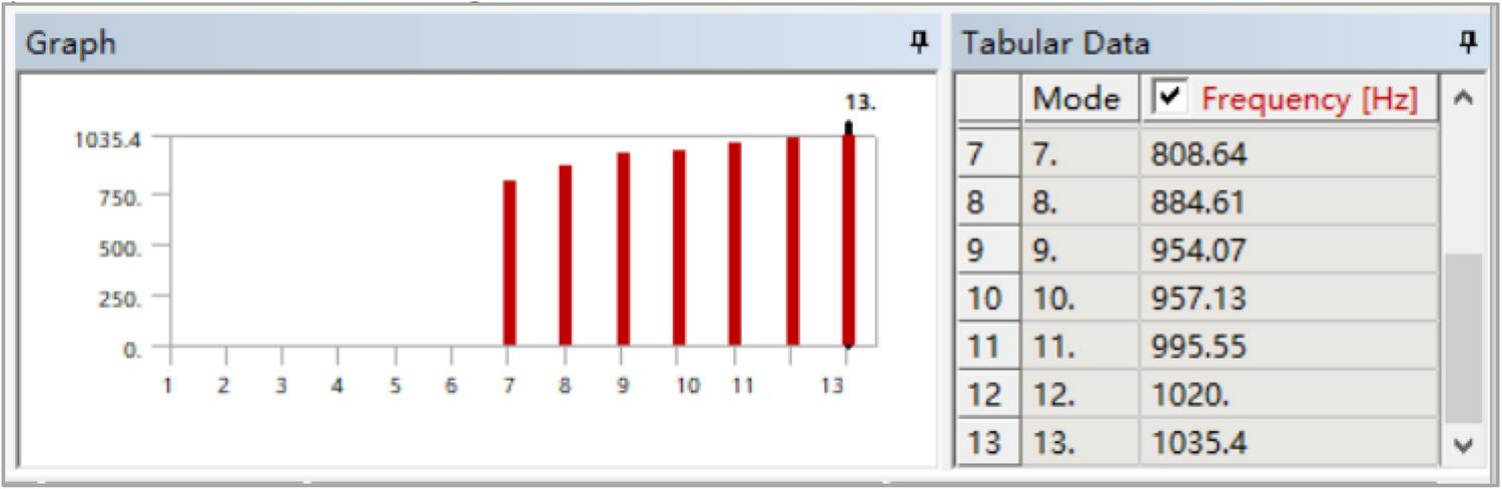
Natural frequencies of the updated box.

Based on the updated material parameters and geometric parameters, the finite element model of the box is rebuilt, and the first six natural frequencies are obtained by modal analysis, as shown in Fig. 12.

The frequencies calculated by the updated finite element model and those obtained by the test are shown in Table 4. It can be seen from the table that the error of the first five order frequencies after model updating is significantly reduced, while the error of the 6th-order frequency increases slightly.

**Table 4:** Frequencies of the updated modal calculation and modal test.

Order	1	2	3	4	5	6
Calculation (Hz)	808.64	884.61	954.07	957.13	1020.00	1035.40
Test (Hz)	801.50	890.34	934.16	953.25	1006.37	1065.80
Error	0.89%	0.64%	2.13%	0.41%	1.35%	2.85%

### Box substructure modeling

**Figure 13:**
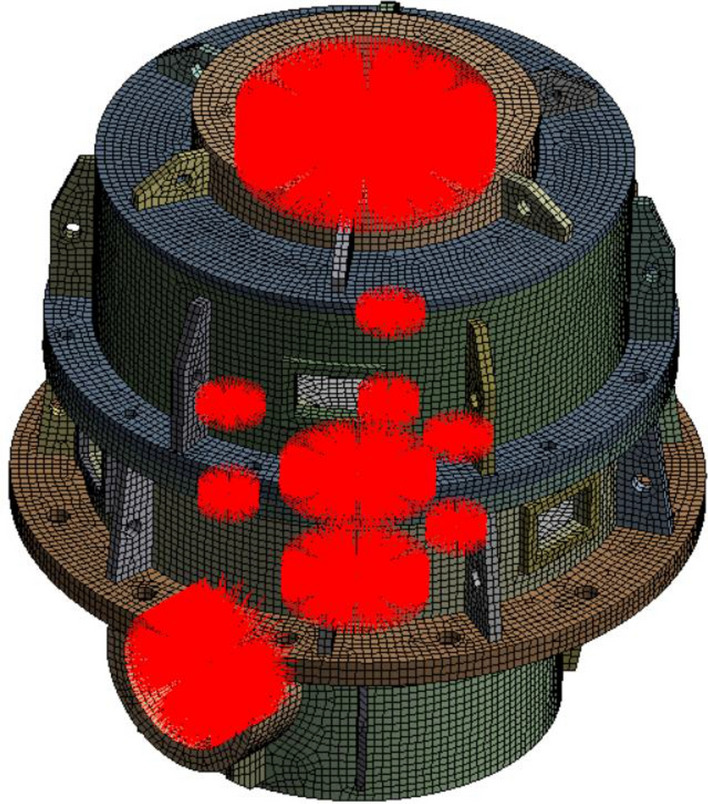
Finite element polycondensation model of the box.

Based on the updated finite element model, the center of the support bearing hole on the box is established as the main node, as shown in Fig. 13. The modal analysis of the box is carried out to dynamically condense the internal degrees of freedom of the box. The stiffness, mass, and damping matrix of the box are extracted at the main nodes to realize order reduction and condensation and finally reduce the degree of freedom.

## Dynamic modeling and analysis of the rotor–stator coupling system of the gearbox

The center of the supporting bearing is defined as the coupling point, and the dynamic equation of the rotor–stator coupling system of the gearbox can be obtained by combining the two substructure equations according to the displacement coordination.

$$ \left[ {\begin{array}{*{20}c} {{\varvec{M}}_{{\text{t}}} } \\ {\varvec{0}} \\ \end{array} \user2{ }\begin{array}{*{20}c} {\varvec{0}} \\ {{\varvec{M}}_{{\text{c}}} } \\ \end{array} } \right]\left[ {\begin{array}{*{20}c} {\user2{\ddot{q}}_{{\text{t}}} } \\ {\user2{\ddot{q}}_{{\varvec{c}}} } \\ \end{array} } \right]\user2{ + }\left[ {\begin{array}{*{20}c} {{\varvec{C}}_{{\text{t}}} } \\ {{\varvec{C}}_{{{\text{ct}}}} } \\ \end{array} \user2{ }\begin{array}{*{20}c} {{\varvec{C}}_{{{\text{tc}}}} } \\ {{\varvec{C}}_{{\text{c}}} } \\ \end{array} } \right]\left[ {\begin{array}{*{20}c} {\dot{\user2{q}}_{{\text{t}}} } \\ {\dot{\user2{q}}_{{\text{c}}} } \\ \end{array} } \right]\user2{ + }\left[ {\begin{array}{*{20}c} {{\varvec{K}}_{{\text{t}}} } \\ {{\varvec{K}}_{{{\text{ct}}}} } \\ \end{array} \user2{ }\begin{array}{*{20}c} {{\varvec{K}}_{{{\text{tc}}}} } \\ {{\varvec{K}}_{{\text{c}}} } \\ \end{array} } \right]\left[ {\begin{array}{*{20}c} {{\varvec{q}}_{{\text{t}}} } \\ {{\varvec{q}}_{{\text{c}}} } \\ \end{array} } \right]\user2{ = }\left[ {\begin{array}{*{20}c} {{\varvec{Q}}_{{\text{t}}} } \\ {{\varvec{Q}}_{{\text{c}}} } \\ \end{array} } \right] $$

where ${\varvec{M}}_{{\text{t}}}$, ${\varvec{C}}_{{\text{t}}}$, and ${\varvec{K}}_{{\text{t}}}$ are the mass, damping, and stiffness matrices of the transmission substructure, respectively. ${\varvec{M}}_{{\text{c}}}$, ${\varvec{C}}_{{\text{c}}}$, and ${\varvec{K}}_{{\text{c}}}$ are the mass, damping, and stiffness matrices of the box substructure. ${\varvec{K}}_{{{\text{tc}}}}$(${\varvec{K}}_{{{\text{ct}}}}$) and ${\varvec{C}}_{{{\text{tc}}}} ({\varvec{C}}_{{{\text{ct}}}}$) are the coupling stiffness and damping matrix of the transmission substructure and box substructure, respectively. ${\varvec{q}}_{{\text{t}}}$ and ${\varvec{q}}_{{\text{c}}}$ are the vibration displacement vectors of the transmission substructure and box substructure, respectively. ${\varvec{Q}}_{{\text{t}}}$ and ${\varvec{Q}}_{{\text{c}}}$ are the load vectors of the transmission substructure and box substructure, respectively. Based on the equation of the coupling system, the vibration response characteristics of the transmission system considering the flexibility of the box can be analysed.

### Vibration response of the transmission system

The load torque of the inner and outer shafts is taken as *T*_r_ = *T*_c_ = 2815 N m, and the input speed is changed. The vibration response of the transmission components is obtained by solving Eqs. (53) and (55) to analyze the influence of the flexibility of the box on the vibration characteristics of the transmission system.

1. Vibration displacement response.

**Figure 14:**
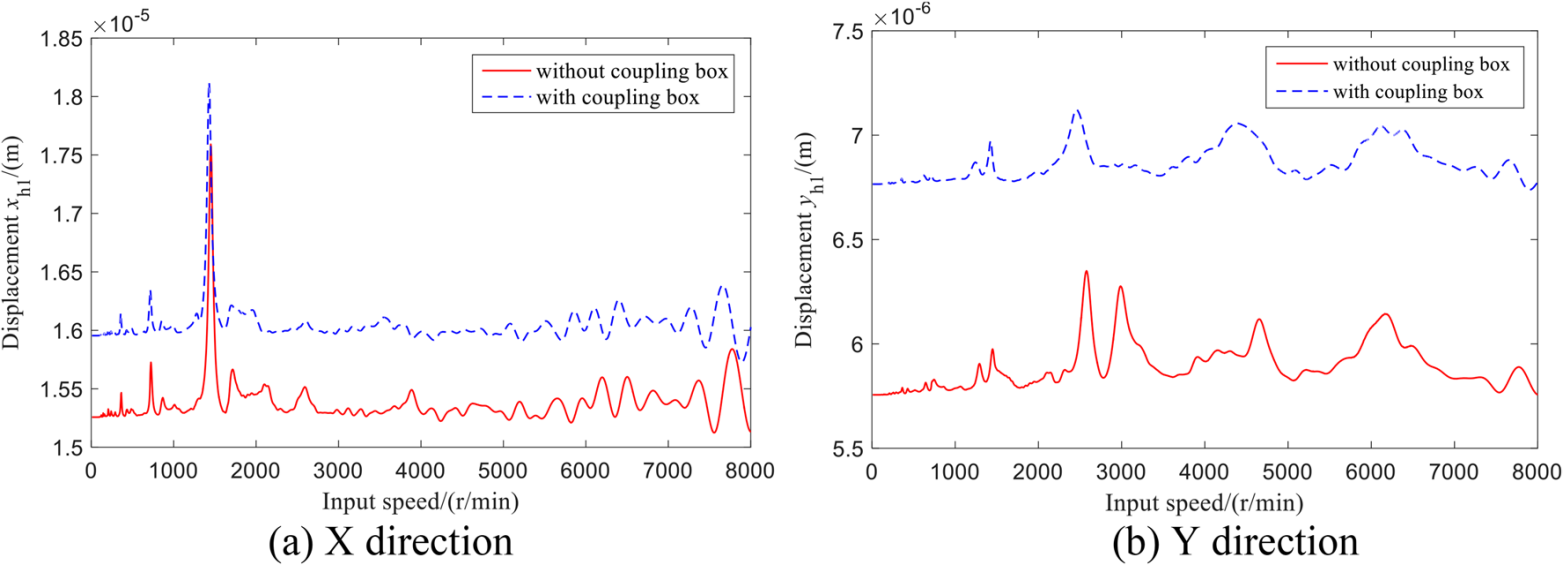
Vibration displacement of the input bevel gear.

The vibration displacement response of the input bevel gear with the rotation speed is shown in Fig. 14. It can be seen from the figure that with the increase in the input rotation speed, the vibration displacement of the input bevel gear in the system with or without coupling box has varying degrees of resonance peaks.

The vibration displacement of the rotor–stator coupling system is larger than that without the coupling box, and the fluctuation range is smaller than that without the coupling box. This is because the overall stiffness of the system is reduced after considering the flexibility of the box, and part of the vibration energy is absorbed, which makes the fluctuations more stable.

**Figure 15:**
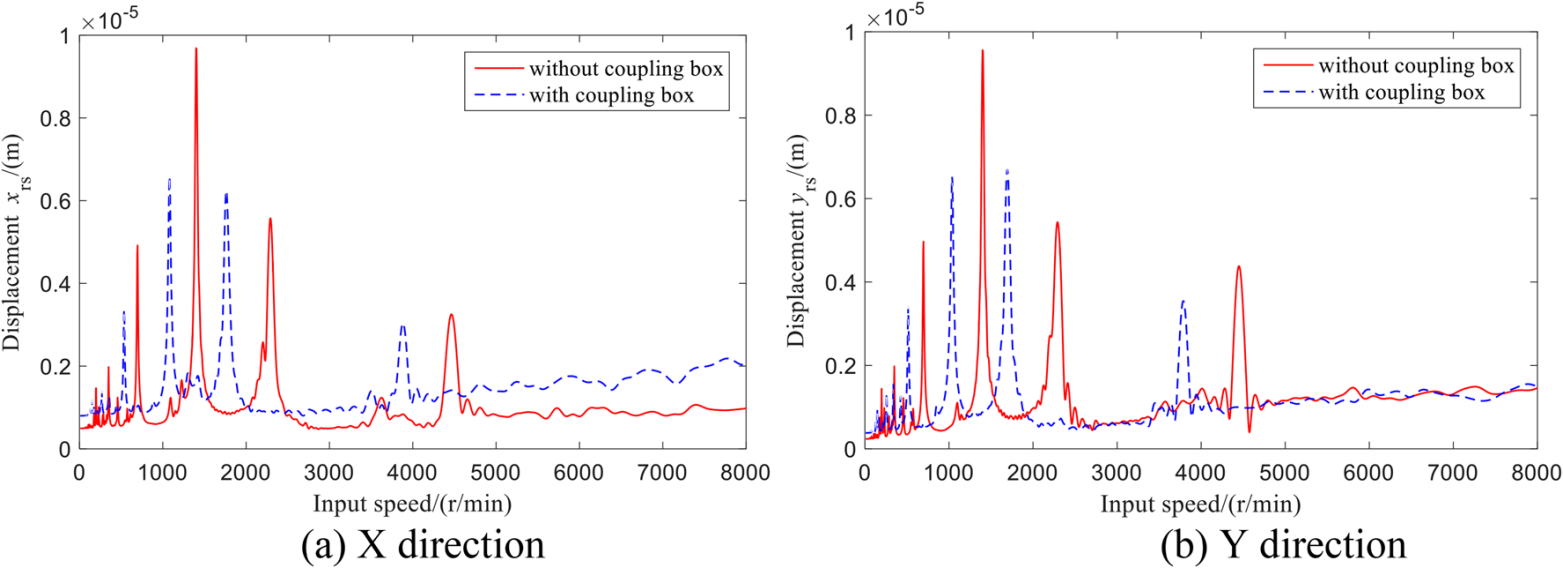
Vibration displacement of the outer output shaft.

The vibration displacement of the outer output shaft is shown in Fig. 15. Similarly, the vibration displacement fluctuation ranges of the rotor–stator coupling system coupling the box are small, and the peak value is offset.

2. Vibration acceleration response.

**Figure 16:**
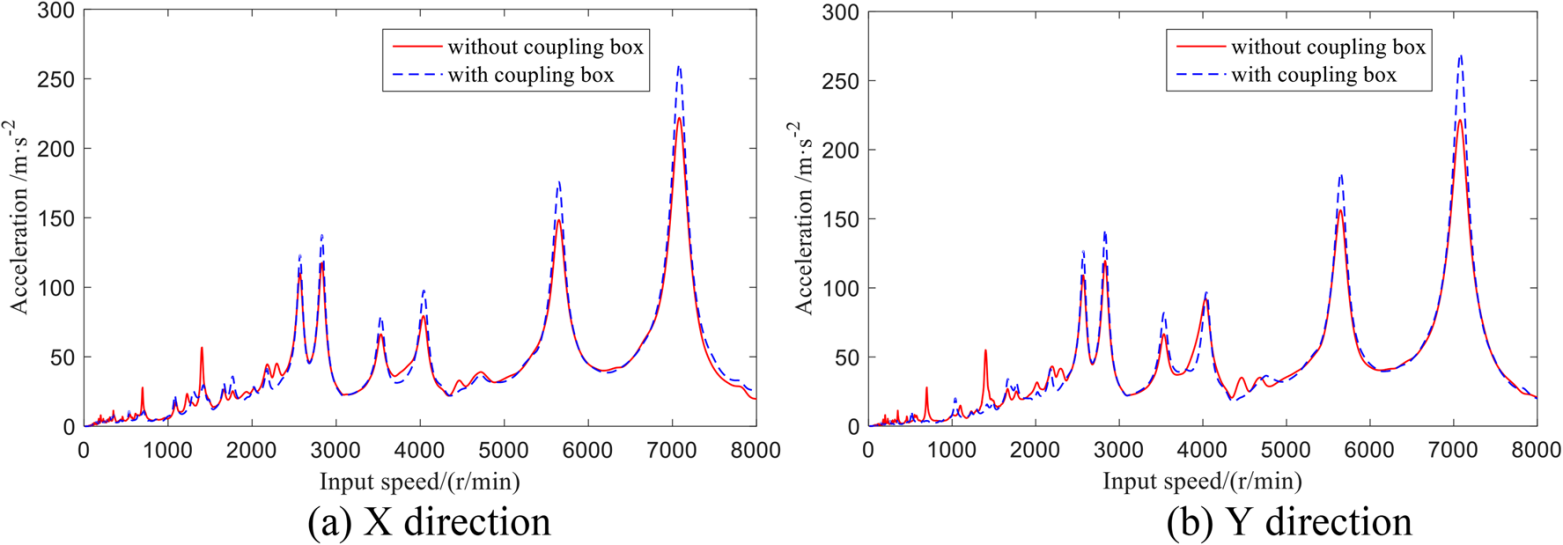
Vibration acceleration of the outer output shaft.

The radial vibration acceleration of the outer output shaft varying with the speed is shown in Fig. 16. At the high-speed resonance point, the acceleration of the shaft of the rotor–stator coupling system is greater than that without coupling the box, while the acceleration at the low-speed resonance point is smaller.

### Vibration response of the box

When the input speed of the gearbox is *n*_in_ = 965.5 r/min, the dynamic supporting force at the bearing of the coupling system is calculated by the following equation.

55 $$ F_{b} \left( t \right) = k_{x} \left( t \right)x_{t} \left( t \right) + c_{t} \left( t \right)\dot{x}_{t} \left( t \right) - k_{c} \left( t \right)x_{c} \left( t \right) - c_{c} \left( t \right)\dot{x}_{c} \left( t \right) $$

where, $k_{t}$, $c_{t}$ are the supporting stiffness and damping of the bearing, and $k_{c}$, $c_{c}$ are the supporting stiffness and damping coefficient of the equivalent node of the box. $x_{t}$,$\dot{x}_{t}$ are the components of dynamic displacement and velocity of bearing nodes in the coordinate axis direction. $x_{c}$, $\dot{x}_{c}$ are the components of the dynamic displacement and velocity of the equivalent node of the box in the coordinate axis direction, respectively.

**Figure 17:**
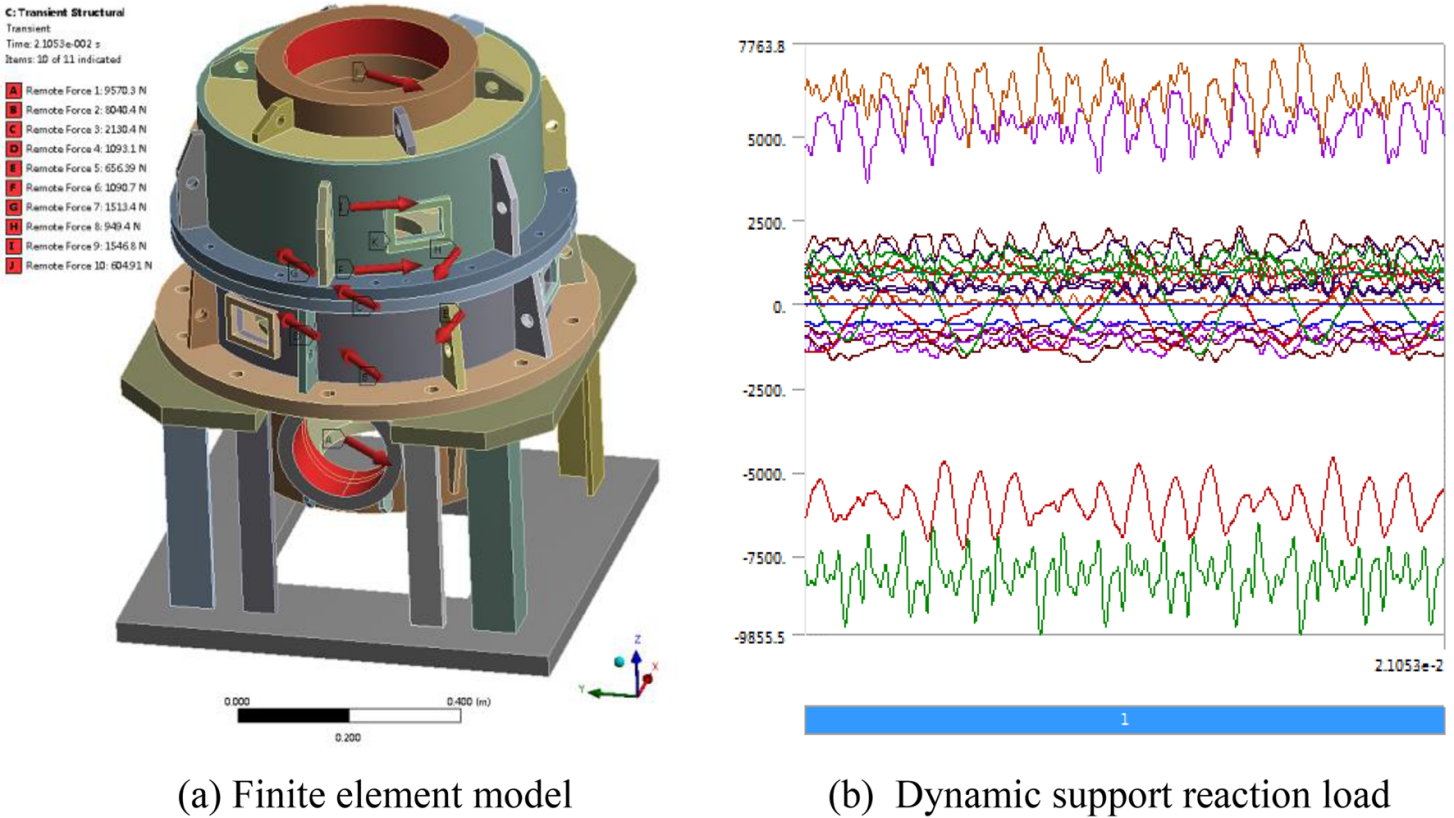
Vibration response analysis model of the box.

The finite element analysis model of the box with dynamic support reaction force as excitation and boundary conditions such as displacement constraints is defined, as shown in Fig. 17. Among them, Fig. 17a shows the point and direction of action of the bearing support reaction force, Fig. 17b shows the component forces along the coordinate direction of each support reaction force, and the label in the horizontal axis direction is time (s), while the label in the vertical axis direction is the magnitude value of the force (N).

**Figure 18:**
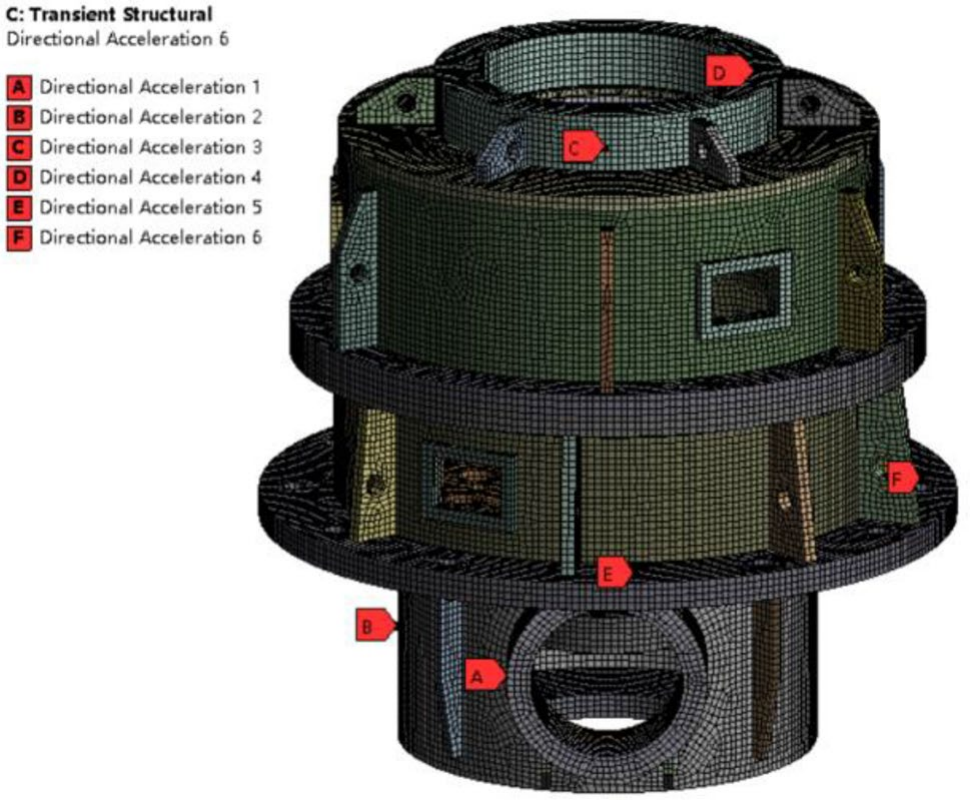
Vibration response analysis model of the box.

The vibration response of the box can be calculated by using the ANSYS transient structure. The finite element simulation calculation is carried out, and the characteristics of the vibration acceleration of the box at the support of the input bevel gear pair and output shaft, as well as the installation position of the gearbox, are analyzed, as shown in Fig. 18.

The root mean square value of normal vibration acceleration at each response point obtained by simulation is shown in Table 5.

**Table 5:** Normal vibration acceleration.

Point	A	B	C	D	E	F
Acceleration (RMS m/s^2^)	13.04	20.56	2.98	6.62	3.27	5.20

It can be seen from Figs. 1, 18 and Table 5 that the vibration response of the box near the support of the input bevel gear pair is the largest, which is due to the large excitation of the box caused by the support reaction force caused by the meshing force of the input stage bevel gear pair, while the vibration response at the support of the output shaft and the installation of the gearbox is small.

## Conclusions

Based on the modal test data, the finite element model of the box is updated. The stiffness matrix, mass matrix, and damping matrix at the main node of the updated box are extracted using the super element method, achieving the reduction and condensation of the box model and reducing the degree of freedom. A coupled dynamic model of the coaxial contra-rotating gearbox rotor–stator system is established using displacement coordination conditions, and the vibration characteristics of the system are analyzed based on this model. The results of this study can be summarized as follows:

1. The optimization method based on the response surface can effectively update the finite element calculation model of the box and reduce the error between the finite element calculation and modal test.
2. With increasing input speed, the vibration response of various components in the system will exhibit resonance peaks to varying degrees whether the box is considered or not. When considering the flexibility of the box, the fluctuation of the component is smaller than that without the box.
3. The results of the vibration response of the box calculated by the transient structure method show that the response of the support of the bevel gear pair is large and that of the output shaft is small.

## Data Availability

The data used to support the findings of this study are available from the corresponding author upon request.
